# Ybp2 Associates with the Central Kinetochore of *Saccharomyces cerevisiae* and Mediates Proper Mitotic Progression

**DOI:** 10.1371/journal.pone.0001617

**Published:** 2008-02-20

**Authors:** Kentaro Ohkuni, Rashid Abdulle, Amy Hin Yan Tong, Charles Boone, Katsumi Kitagawa

**Affiliations:** 1 Department of Molecular Pharmacology, St. Jude Children's Research Hospital, Memphis, Tennessee, United States of America; 2 Banting and Best Department of Medical Research, University of Toronto, Toronto, Canada; 3 Department of Medical Genetics and Microbiology, University of Toronto, Toronto, Canada; Fred Hutchinson Cancer Research Center, United States of America

## Abstract

The spindle checkpoint ensures the accurate segregation of chromosomes by monitoring the status of kinetochore attachment to microtubules. Simultaneous mutations in one of several kinetochore and cohesion genes and a spindle checkpoint gene cause a synthetic-lethal or synthetic-sick phenotype. A synthetic genetic array (SGA) analysis using a *mad2*Δ query mutant strain of yeast identified *YBP2*, a gene whose product shares sequence similarity with the product of *YBP1*, which is required for H_2_O_2_-induced oxidation of the transcription factor Yap1. *ybp2Δ* was sensitive to benomyl and accumulated at the mitotic stage of the cell cycle. Ybp2 physically associates with proteins of the COMA complex (Ctf19, Okp1, Mcm21, and Ame1) and 3 components of the Ndc80 complex (Ndc80, Nuf2, and Spc25 but not Spc24) in the central kinetochore and with Cse4 (the centromeric histone and CENP-A homolog). Chromatin-immunoprecipitation analyses revealed that Ybp2 associates specifically with *CEN* DNA. Furthermore, *ybp2Δ* showed synthetic-sick interactions with mutants of the genes that encode the COMA complex components. Ybp2 seems to be part of a macromolecular kinetochore complex and appears to contribute to the proper associations among the central kinetochore subcomplexes and the kinetochore-specific nucleosome.

## Introduction

The centromere interacts with the spindle fibers to ensure the correct segregation of chromosomes during mitotic and meiotic cell divisions. It requires DNA sequence elements and structural and regulatory proteins for its activity and coordination within the cell cycle. Kinetochores are specialized protein complexes that assemble on the centromeric (*CEN*) DNA and mediate the attachment of duplicated chromosomes to the mitotic spindle; they also generate signals to arrest cell cycle progression if metaphase is not achieved properly (reviewed in [1], [2]). After spindle pole body duplication, sister centromeres separate transiently and oscillate along the spindle axis until anaphase, when they separate permanently [3]–[6].

The budding yeast *Saccharomyces cerevisiae* has short 125-bp point centromeres and its kinetochores bind to single microtubules. The kinetochore consists of 3 protein layers (inner, central, and outer) that assemble hierarchically onto the *CEN* DNA (reviewed in [7]–[9]). The inner kinetochore proteins are in direct contact with the *CEN* DNA. In *S. cerevisiae*, the inner kinetochore consists of the CBF3 complex and a specialized nucleosome containing the conserved histone H3-like protein Cse4 (Cnp1 in fission yeast and CENP-A in higher eukaryotes). The CBF3 complex is composed of 4 essential proteins: Ndc10/Ctf14/Cbf2, Cep3/Cbf3b, Ctf13, and Skp1. The inner kinetochore complex binds to *CEN* DNA and is required for the centromeric association of all other kinetochore proteins. The central kinetochore consists of at least 3 major subcomplexes: the COMA complex (Ctf19, Okp1, Mcm21, and Ame1); the MIND complex (Mtw1, Nnf1, Nsl1, and Dsn1); and the Ndc80 complex (Ndc80, Nuf2, Spc24, and Spc25). The central kinetochore complex connects the inner kinetochore to various microtubule-binding proteins. The outer kinetochore consists of the Dam1 complex, which contains 9 or more subunits. The outer kinetochore proteins associate with microtubules for their kinetochore association. Recent studies have implicated the conserved Ndc80 complex in several essential outer kinetochore functions, including microtubule binding and control of a safety device known as the spindle checkpoint [10]–[13].

In addition to kinetochore proteins, numerous other proteins are integral to chromosome stability, including spindle checkpoint proteins, motor proteins, microtubule-associated proteins, regulatory proteins, and proteins implicated in *CEN* DNA chromatin dynamics, structure, and sister chromatid cohesion [1], [7], [14], [15]. Proteomic approaches have identified structural components of the kinetochore, and genetic approaches have identified various proteins important for chromosome segregation in yeast. For instance, a chromosome transmission fidelity (CTF) screen has identified mutations in genes encoding DNA replication, cohesion, and kinetochore proteins [16]. Synthetic dosage lethal (SDL) screens in which mutants that cannot tolerate overexpression of kinetochore proteins have identified chromosome stability genes, many of which are not components of the kinetochore (e.g., chromatin-modifying or tubulin-folding proteins) [17]–[23]. Synthetic genetic array (SGA) analysis has enabled investigators to perform systematic genome-wide genetic screens in yeast [24].

The spindle checkpoint ensures accurate chromosomal segregation by monitoring that anaphase is initiated only after proper kinetochore–microtubule associations of all sister chromatids occur. It thereby safeguards against missegragation events during mitosis and meiosis; recent studies have shown that spindle checkpoint dysfunction might facilitate tumor progression (reviewed in [25], [26]). Chief components of the spindle checkpoint complex in *S. cerevisiae* include the mitotic arrest–defective (*MAD*) genes *MAD1–3* [27] and the budding-uninhibited-by-benzimidazole (*BUB*) genes *BUB1* and *BUB3* [28]. Simultaneous mutations in one of several kinetochore and cohesion genes, such as *CTF19* and *CTF8*, and a spindle checkpoint gene cause a synthetic-lethal or synthetic-sick phenotype [29]–[31]. Thus, a synthetic lethality screen that uses a spindle checkpoint mutant should identify genes whose functions are monitored by the spindle checkpoint. We therefore characterized the mitotic function of Ybp2 that was identified by an SGA screen by using the *mad2*-deletion (*mad2*Δ) strain.

## Results

### Synthetic Genetic Array (SGA) Screen of *mad2*Δ Mutants Identified *YBP2* as a Mitotic Factor

To identify genes that regulate mitosis in yeast, an SGA screen of the *mad2*Δ strain as a query mutation was performed against a complete set of deletion-mutant yeast strains [24]. SGA allows crossing of the query mutation into the set of viable deletion mutants, thereby allowing the resulting double mutants to be screened for synthetic-lethal or synthetic-sick interactions. The *mad2*Δ SGA screen detected previously described kinetochore proteins, microtubule-binding proteins, chromatin proteins, and cohesion proteins [24]. Mutants of most known nonessential outer and central kinetochore genes were synthetic-lethal or synthetic-sick when combined with the *mad2*Δ mutation, confirming that the screen worked as expected. Of the 32 genes detected, 4 have not been previously identified or characterized as mitotic factors. One of them has recently been named Ybp2 because of its homology with Ybp1 (Yap1-binding protein 1), a protein required for oxidation of the transcription factor Yap1 in response to H_2_O_2_ [32].

**Figure 1:**
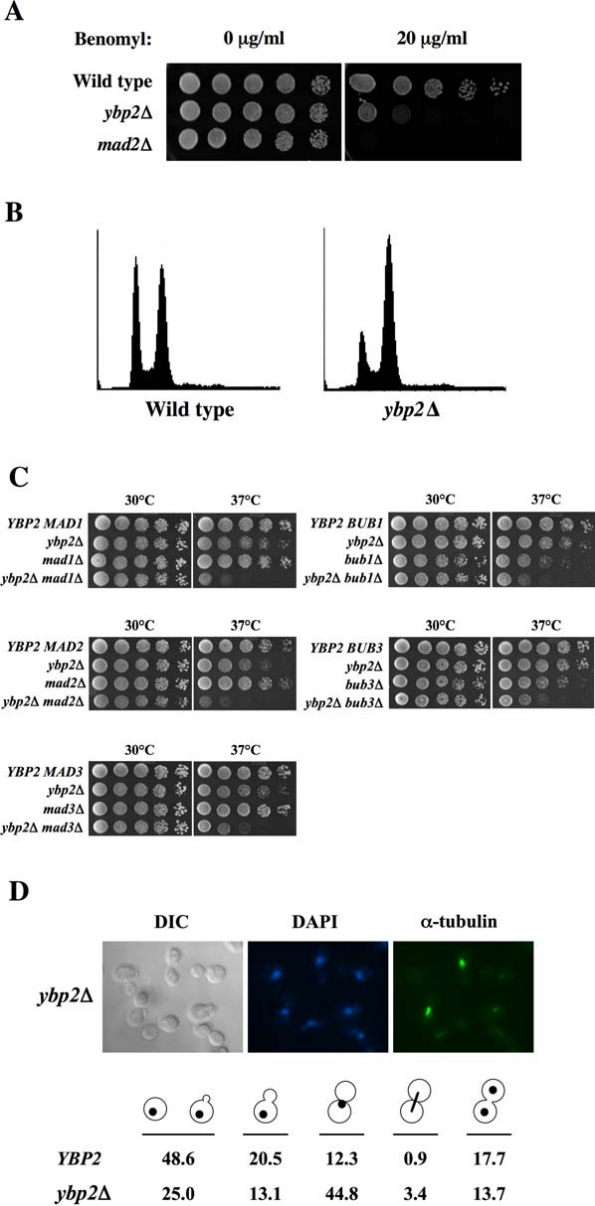
Ybp2 has a mitotic function. (A) The *ybp2*Δ mutant is sensitive to benomyl. Wild-type (BY4741), *ybp2*Δ (Y1028), and *mad2*Δ (Y516) strains were spotted in 5-fold dilutions from 5×10^4^ cells per spot on YPD plates containing 20 µg/mL benomyl and incubated at 25°C for 3 days. (B) The *ybp2*Δ cells accumulate at G2/M. Logarithmically growing cells, wild type (Y14) and *ybp2*Δ (Y1831), were cultured at 25°C and processed for flow cytometry. (C) Double mutants of *ybp2*Δ and the spindle checkpoint genes show temperature-dependent synthetic lethality. Yeast strains were spotted in 5-fold dilutions from 5×10^4^ cells per spot on YPD plates. The plates were incubated at the indicated temperatures for 2 days. Isogenic yeast strains were wild type (YPH500), *ybp2*Δ (Y1342), *mad1*Δ (Y550), *mad2*Δ (Y1323), *mad3*Δ (Y554), *bub1*Δ (Y530), *bub3*Δ (Y548), *ybp2*Δ*mad1*Δ (Y1398), *ybp2Δmad2*Δ (Y1419), *ybp2*Δ*mad3*Δ (Y1402), *ybp2*Δ*bub1*Δ (Y1415), and *ybp2*Δ*bub3*Δ (Y1396). (D) Immunofluorescence analysis of *ybp2*Δ cells. Logarithmically growing cells cultured at 25°C were fixed and stained with antitubulin antibodies and DAPI. The strains shown are wild type (Y14) and *ybp2*Δ (Y1831). For each sample, 300 cells were counted.

We found that the *ybp2Δ* strain was sensitive to benomyl (Figure 1A) and accumulated at the G2/M stage of the cell cycle (Figure 1B), traits common to most kinetochore mutants. These results suggest that *YBP2* is required for mitotic function.

### Phenotypic Analysis of the *ybp2*Δ Mutant

To further examine the potential synthetic lethality of *ybp2*Δ with *mad2*Δ, we constructed a *ybp2*Δ*mad2*Δ double mutant carrying the *MAD2 CEN-URA3* plasmid. We used the 5-fluoroorotic acid (FOA) that selects against strains that contain the *URA3* gene. The double mutant grew on a 5-FOA plate (unpublished data), indicating that the interaction between *YBP2* and *MAD2* is not a straightforward synthetic-lethal interaction. Next, we constructed a *ybp2*Δ*mad2*Δ double mutant in our background (YPH499) to assess the double mutant for a more subtle synthetic fitness defect. The *ybp2*Δ*mad2*Δ double-mutant cells grew relatively normally at 30°C but failed to grow at 37°C (Figure 1C). The double mutants of *ybp2*Δ and other spindle checkpoint mutants (*ybp2*Δ*bub1*Δ, *ybp2*Δ*mad1*Δ, *ybp2*Δ*mad3*Δ, and *ybp2*Δ*bub3*Δ) showed the same temperature sensitivity (Figure 1C). Thus, *YBP2* has a temperature-sensitive conditional synthetic-lethal interaction with spindle checkpoint genes.

**Figure 2:**
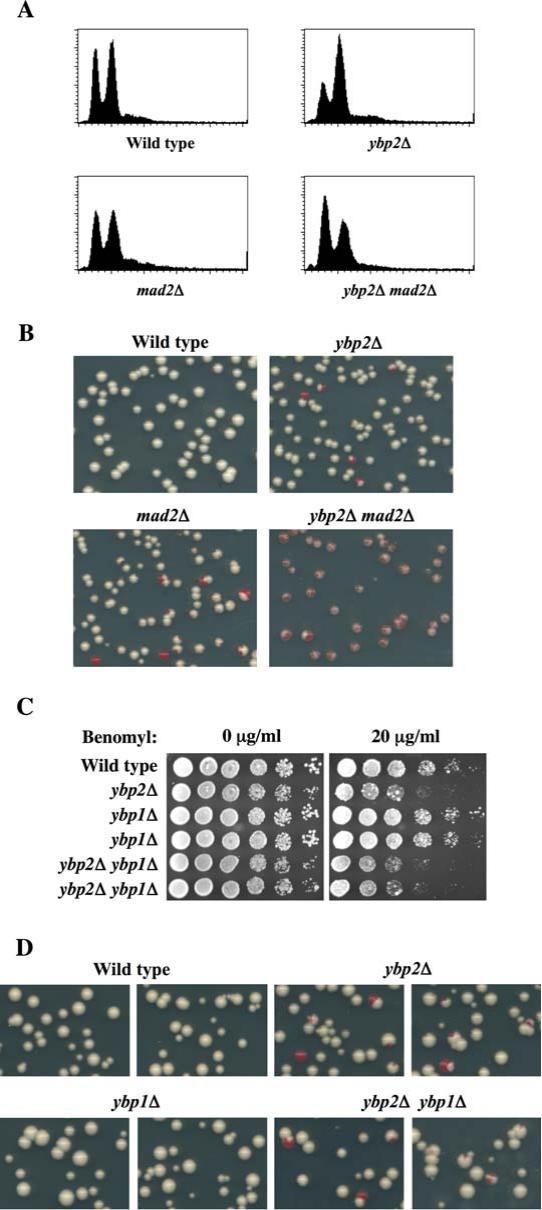
The mitotic defect of the *ybp2Δ* mutant is enhanced by *mad2Δ* but not *ybp1Δ*. (A) The G2/M delay of *ybp2*Δ cells depends on *MAD2*. Logarithmically growing cells cultured at 25°C were processed for flow cytometry. (B) The *ybp2*Δ strain does not display a substantial chromosome-missegregation phenotype. Chromosome fragment segregation was analyzed by a colony color assay (see Materials and Methods). Loss of nonessential chromosome fragments results in a red sector in a white colony. Isogenic yeast strains used in (A) and (B) were wild type (Y14), *ybp2*Δ (Y1831), *mad2*Δ (Y1833), and *ybp2*Δ*mad2*Δ (Y1834). (C) The phenotype of the *ybp1*Δ mutant was not similar to that of the *ybp2*Δ mutant. Yeast strains were spotted in 5-fold dilutions from 5×10^4^ cells per spot on YPD plates containing the indicated concentrations of benomyl and incubated at 30°C for 2–3 days. (D) The *ybp2*Δ cells showed a subtle chromosome-missegregation phenotype, but *ybp1*Δ cells did not. The *ybp2*Δ*ybp1*Δ double mutant exhibited the same subtle chromosome-missegregation phenotype, indicating that Ybp1 does not participate in the mitotic function of Ybp2. Isogenic yeast strains used in (C) and (D) were wild type (Y14), *ybp2*Δ (Y1831), *ybp1*Δ (Y1835), and *ybp2*Δ*ybp1*Δ (Y1836).

The *ybp2*Δ mutant arrested with largely G2/M DNA content in haploid cells containing a nonessential marked chromosome fragment (Figure 1B) and in diploid cells (Supplemental Figure S1), but, surprisingly, not in normal haploid cells that did not contain an extra chromosome fragment (unpublished data). Quantification of cell and nuclear morphology in haploid *ybp2*Δ cells containing the chromosome fragment revealed that ∼45% of the cells were large budded, with an undivided nucleus positioned at or near the neck between the mother and daughter cells (Figure 1D). The G2/M delay caused by *ybp2*Δ was abolished in the absence of Mad2 (Figure 2A), which indicates that the G2/M delay is dependent on the spindle checkpoint.

We examined chromosome stability in *ybp2*Δ mutant cells by a colony color assay, which measures the stability of a marker chromosome fragment [33]. The *ybp2Δ* mutant cells showed only a moderate chromosome-missegregation phenotype (chromosome fragment loss 1.0%) (Figures 2B and D and Supplemental Figure S2). However, *ybp2*Δ*mad2*Δ exhibited an enhanced chromosome-missegregation phenotype (chromosome fragment loss: 42%) as compared with either single mutant (Figure 2B). These results suggest that the mitotic defect caused by deletion of *YBP2* is monitored by the spindle checkpoint, and thus the *ybp2*Δ cells are protected from chromosome missegregation.

The synthetic lethal screen is generally employed to identify genes encoding proteins that function in a parallel pathway or with overlapping functions within an essential pathway. Because we used the spindle checkpoint mutant *mad2*Δ as a query, we examined whether Ybp2 functions in the spindle checkpoint. A major phenotype of spindle checkpoint mutants is repeated budding without mitotic arrest in the presence of the microtubule-depolymerizing drug nocodazole. However, *ybp2*Δ mutant cells arrested in the G2/M phase of the cell cycle (Supplemental Figure S3A), indicating that the spindle checkpoint is activated in response to unattached kinetochores.

Ctf8 is required for proper sister chromatid cohesion [30]. In *ctf8* mutants, microtubules can attach to kinetochores but the tension at kinetochores is reduced because the linkage between sister chromatids is compromised. We arrested *ctf8*Δ, *ctf8*Δ*ybp2*Δ, and wild-type cells in G1 with α-factor at 30°C and then released them into a YPD medium without α-factor. There was a delay in Pds1 degradation in *ctf8*Δ*ybp2*Δ and *ctf8*Δ cells, indicating spindle checkpoint activation (Supplemental Figure S3B). These results indicate that Ybp2 is not required for spindle checkpoint function.

In addition, we found that *ybp2Δ* cells did not exhibit sister chromatid cohesion defects by the cohesion assay, using a strain an array of lactose operators integrated at the *TRP1* locus, 12 kb from the *CEN* of chromosome IV and expressing a GFP-lactose repressor (GFP-lacI) [34], [35] (Supplemental Figure S4).

### Ybp1 does not Have a Mitotic Function

Recent studies suggest that Ybp2, which is homologous (35% identity) to Ybp1, might be required for oxidative stress tolerance because Ybp1 is a positive regulator of oxidative stress tolerance [32], [36]. Therefore, we examined whether Ybp1 is also required for a mitosis function. The *ybp1*Δ mutant cells showed no benomyl sensitivity, and there was no enhanced benomyl sensitivity in the *ybp2*Δ*ybp1*Δ double mutant (Figure 2C). Also, *ybp1*Δ cells did not show a chromosome-missegregation phenotype, and deletion of *YBP1* did not enhance the missegregation phenotype of *ybp2*Δ*ybp1*Δ cells (Figure 2D). These results suggest that Ybp1 does not play a significant role in the mitotic function of Ybp2.

### Ybp2 Interacts with Several Central Kinetochore Proteins and the Centromeric Histone Cse4

**Figure 3:**
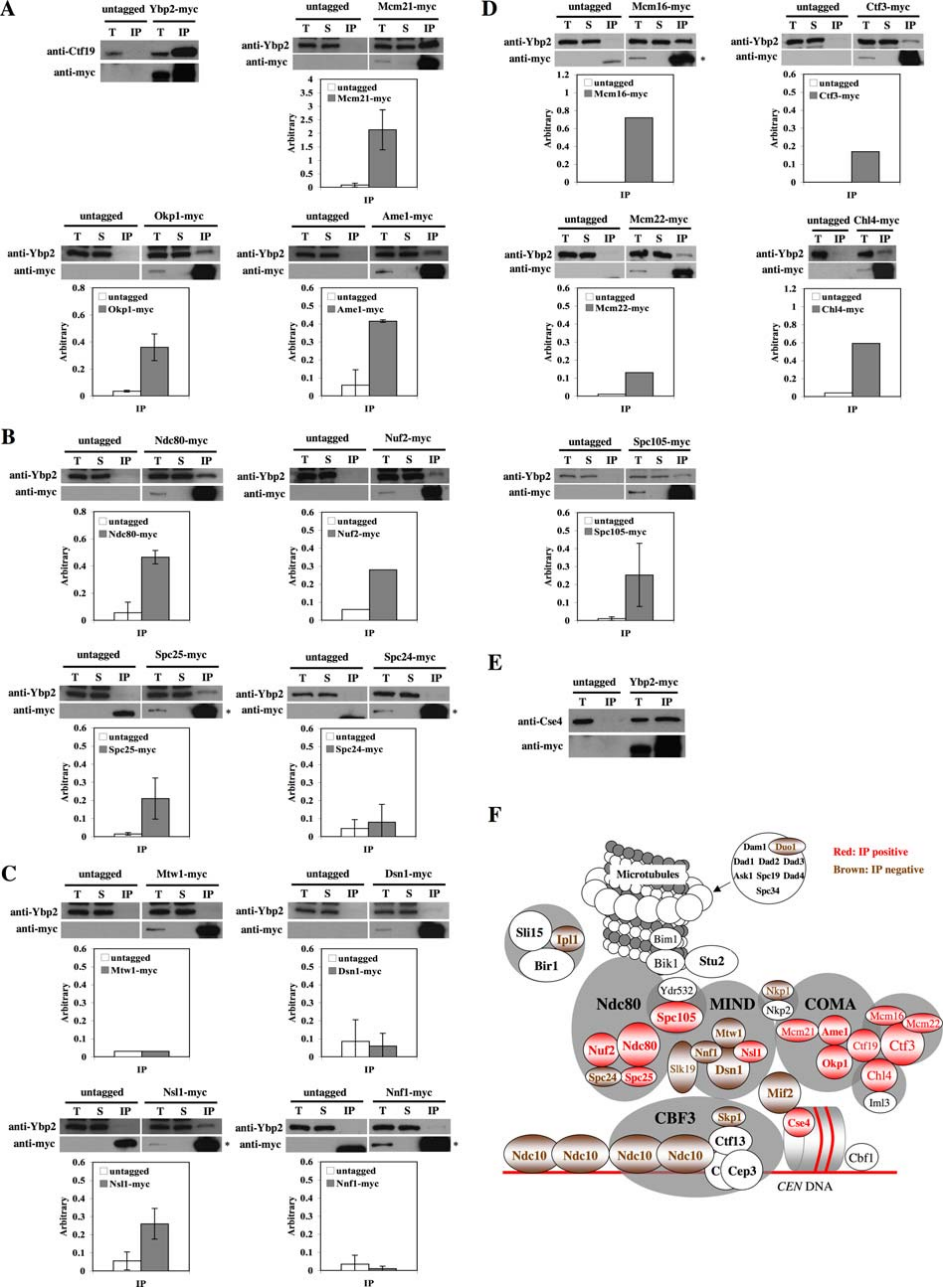
Ybp2 physically associates with the COMA and Ndc80 complexes and Cse4. (A–E) The indicated strains were grown to log phase at 30°C, lysed, and anti-myc immunoprecipitations were performed. Total lysate (T), supernatant (S), and the immunoprecipitated fraction (IP) were subjected to SDS-PAGE, and Western blots were used to detect Ybp2, Ctf19, Cse4, or myc-tagged proteins with the respective antibodies. For the quantification of Ybp2, aliquots of the total lysate (1/500 of the total), supernatant (1/500 of the total), and IP fraction (2/5 of the total lysate) were loaded. Arbitrary number is defined as the ratio of the amount of coprecipitated protein to the amount of input protein. Intensity of the total lysate is defined as 1 in each strain. Error bars indicate standard deviations of 2 or 3 experiments. The yeast strains used were (A) untagged (YPH499), Ybp2-myc (Y1689), Okp1-myc (Y1707), Mcm21-myc (Y1708), and Ame1-myc (Y1706); (B) untagged (YPH499), Ndc80-myc (Y1713), Nuf2-myc (Y1714), Spc24-myc (Y1715), and Spc25-myc (Y1716); (C) untagged (YPH499), Mtw1-myc (Y1709), Nsl1-myc (Y1710), Nnf1-myc (Y1711), and Dsn1-myc (Y1712); (D) Mcm16-myc (YVM325), Mcm22-myc (YVM290), Ctf3-myc (YVM219), Spc105-myc (Y1717), and Chl4-myc (YPH1542); and (E) untagged (YPH499) and Ybp2-myc (Y1689). Asterisks indicate IgG bands in immunoprecipitates. (F) Summary of coimmunoprecipitation assay results. Several kinetochore proteins were detected (red) in the Ybp2 immune complexes and others were not (brown).

**Figure 4:**
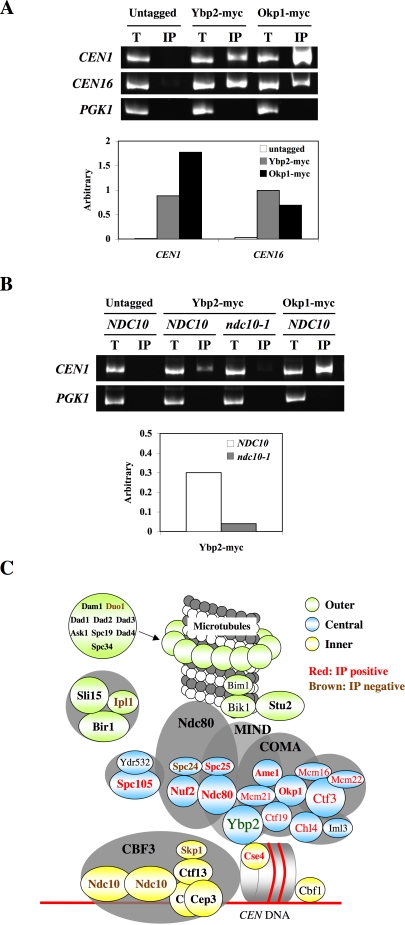
Ybp2 associates with *CEN* DNA in an Ndc10-dependent manner. (A and B) Anti-myc chromatin immunoprecipitation (ChIP) assays were performed from log-phase cells at 30°C (A) or 25°C (B). Total lysate (T, 1/800 of the total) and coimmunoprecipitated DNA (IP, 1/10 of the total) were analyzed by PCR with primers specific to centromeric regions of chromosome I and XVI and to a noncentromeric region (*PGK1*) as a control for binding specificity. The myc-tagged Okp1 strain was used as positive control. For the quantification of *CEN1* and *CEN16*, aliquots of the total lysate (2/25 of the total) and IP fraction (2/5 of the total) were loaded on an 8% acrylamide gel. Arbitrary number is defined as the ratio of the amount of coprecipitated DNA to the amount of input DNA. Intensity of the total lysate is defined as 1 in each strain. The yeast strains used were (A) untagged (YPH499), Ybp2-myc (Y1689), and Okp1-myc (Y1707); and (B) untagged (YPH499), Ybp2-myc (Y1689), Ybp2-myc *ndc10-1* (Y1838), and Okp1-myc (Y1707). (C) A model of the role of Ybp2 in the central kinetochore. Ybp2 interacts strongly with proteins in the COMA and Ndc80 complexes (shown in red) but not as well with those in the MIND complex and other kinetochore proteins (shown in brown). We propose that Ybp2 is localized to bridge the COMA and Ndc80 complexes onto the centromeric nucleosome.

To examine the interaction between Ybp2 and known kinetochore proteins, we generated antibodies against Ybp2 and strains that express several myc-tagged kinetochore proteins under their own promoters. We also confirmed that all tagged proteins were functional (unpublished data). Ybp2 or Ybp2-myc coimmunoprecipitated with all components of the COMA complex (Ctf19, Okp1, Mcm21, and Ame1; Figure 3A); 3 of 4 components of the Ndc80 complex (Ndc80, Nuf2, and Spc25 but not Spc24; Figure 3B); and 1 of 4 components of the MIND complex (Nsl1 but not Mtw1, Nnf1, or Dsn1; Figure 3C). Ybp2 coimmunoprecipitated with Ctf3, Mcm16, Mcm22, Chl4, and Spc105 (Figure 3D). Interestingly, Ybp2-myc interacted with Cse4, the centromeric histone H3 (yeast CENP-A homolog) (Figure 3E). Ybp2 did not coimmunoprecipitate with Ipl1, Slk19, Mif2, Ndc10 (Supplemental Figure S5), Duo1, Nkp1, or Skp1 (unpublished data). These results indicate that Ybp2 associates with the central kinetochore complexes, especially the COMA and Ndc80 complexes, and the centromeric nucleosomes (Figure 4G).

Because previous chromatin-immunoprecipitation (ChIP) assays have shown that all the central kinetochore proteins characterized associate with *CEN* DNA [1], we performed ChIP assays by using Ybp2-myc strains. Myc-tagged Ybp2 was immunoprecipitated from cross-linked extracts with anti-myc–conjugated beads. The coimmunoprecipitated DNA was analyzed by PCR with primers specific to centromeric regions of chromosomes I and XVI and to the noncentromeric region *PGK1* as a control for binding specificity. The Okp1 myc-tagged strain was used as positive control. Ybp2 coimmunoprecipitated specifically with *CEN* DNA (Figure 4A and Supplemental Figure S6A), indicating that it associates with *CEN* DNA in vivo. This association was abolished in *ndc10-1* mutant cells (Figure 4B and Supplemental Figure S6B), indicating that the association of Ybp2 with *CEN* DNA is dependent on Ndc10 and thereby the CBF3 complex. These results reveal that Ybp2 is part of the macromolecular kinetochore complex.

We proposed a model that summarizes the immunoprecipitation and ChIP results (Figure 4C). Ybp2 interacts with proteins in the COMA and Ndc80 complexes, but does not substantially interact with those in the MIND complex. Therefore, these 3 complexes appear to have a 3-dimensional surface. We hypothesized that Ybp2 might be localized among the 3 complexes (Figure 4C) to bridge COMA and Ndc80 onto the centromeric nucleosome.

### Interactions among the 3 Central Kinetochore Subcomplexes Are Increased in *ybp2*Δ Mutant Cells

**Figure 5:**
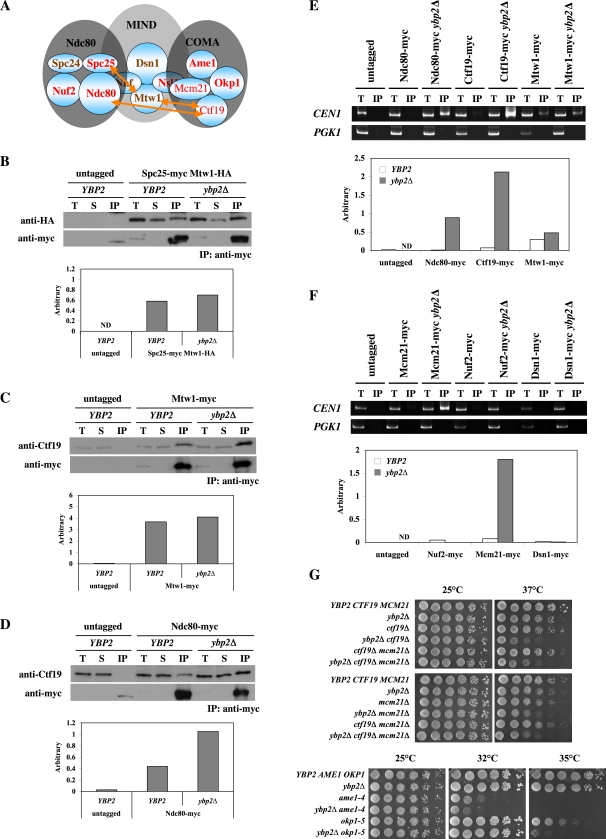
Interactions among 3 central kinetochore subcomplexes in the *ybp2*Δ mutant. (A) The model indicates the positions of the 3 central kinetochore subcomplexes Ndc80, MIND, and COMA. Orange arrows show protein–protein interactions that link the subcomplexes. (B–D) The indicated strains were grown to log phase at 30°C, lysed, and anti-myc immunoprecipitations were performed. Total lysate (T), supernatant (S), and the immunoprecipitated fraction (IP) were subjected to SDS-PAGE, and Western blots were used to detect Ctf19, HA-tagged, or myc-tagged proteins with the respective antibodies. For the quantification of (B) Mtw1-HA, aliquots of the total lysate (1/500 of the total), supernatant (1/500 of the total), and IP fraction (2/25 of the total lysate) were loaded. ND, not detectable. For the quantification of (C) Ctf19, aliquots of the total lysate (1/500 of the total), supernatant (1/500 of the total), and IP fraction (2/5 of the total lysate) were loaded. For the quantification of (D) Ctf19, aliquots of the total lysate (1/1000 of the total), supernatant (1/1000 of the total), and IP fraction (1/5 of the total lysate) were loaded. Arbitrary number is defined as described in Figure 3. The yeast strains used were (B) untagged (YPH499), Spc25-myc Mtw1-HA (Y1837), and Spc25-myc Mtw1-HA *ybp2*Δ (Y1839); (C) untagged (YPH499), Mtw1-myc (Y1709), and Mtw1-myc *ybp2*Δ (Y1840); and (D) untagged (YPH499), Ndc80-myc (Y1713), and Ndc80-myc *ybp2*Δ (Y1841). (E and F) Ndc80, Ctf19, and Mcm21 bind more tightly to *CEN* DNA in the absence of Ybp2. ChIP assays were performed with nocodazole-arrested cells (treated with 20 µg/mL nocodazole for 3 h at 25°C). Total lysate (T, 1/800 of the total) and coimmunoprecipitated DNA (IP, 1/10 of the total) were analyzed by PCR with primers specific to the centromeric regions of chromosome I and to a noncentromeric region (*PGK1*) as a control for binding specificity. For the quantification of *CEN1*, aliquots of the total lysate (2/25 of the total) and IP fraction (2/5 of the total) were loaded in 8% acrylamide gel. Arbitrary number is defined as described in Figure 4. ND, not done. Isogenic yeast strains were (E) untagged (YPH499), Ndc80-myc (Y1713), Ndc80-myc *ybp2*Δ (Y1841), Ctf19-myc (IPY313), Ctf19-myc *ybp2*Δ (Y1842), Mtw1-myc (Y1709), and Mtw1-myc *ybp2*Δ (Y1840); and (F) untagged (YPH499), Mcm21-myc (Y1708), Mcm21-myc *ybp2*Δ (Y1866), Nuf2-myc (Y1714), Nuf2-myc *ybp2*Δ (Y1867), Dsn1-myc (Y1712), and Dsn1-myc *ybp2*Δ (Y1868). (G) Synthetic-sick interaction between *ybp2*Δ and mutation of COMA complex genes. Yeast strains were spotted in 5-fold dilutions from 5×10^4^ cells per spot on YPD plates. The plates were incubated at the indicated temperatures for 2 days. Isogenic yeast strains used were wild type (YPH499), *ybp2*Δ (Y1337), *ctf19*Δ (YPH1315), *mcm21*Δ (Y1824), *ame1-4* (YPH1676), *okp1-5* (YPH1678), *ybp2*Δ*ctf19*Δ (Y1826), *ybp2*Δ*mcm21*Δ (Y1827), *ctf19*Δ*mcm21*Δ (Y1828), *ybp2*Δ*ame1-4* (Y1864), *ybp2*Δ*okp1-5* (Y1865), and *ybp2*Δ*ctf19*Δ*mcm21*Δ (Y1829).

Given the interaction of Ybp2 with the central kinetochore subcomplexes (COMA, MIND, and Ndc80), we expected that Ybp2 might be important for the interaction between the Ndc80 and the COMA complexes (Figure 4C). As per this hypothesis, the Ndc80–COMA association would be disrupted in the *ybp2*Δ background but the Ndc80–MIND and the COMA–MIND associations would not (Figure 5A). To test this hypothesis, we performed coimmunoprecipitation assays using *ybp2*Δ mutant cells. The Spc25–Mtw1 interaction for the Ndc80–MIND association, the Mtw1–Ctf19 interaction for the MIND–COMA association, and the Ndc80–Ctf19 interaction for the Ndc80–COMA association (Figure 5A) have been previously reported [37], [38]. First, we tested the interaction between Spc25 in the Ndc80 complex and Mtw1 in the MIND complex. We constructed an Spc25-myc Mtw1-HA double-tagged strain in the wild-type or *ybp2*Δ strain, performed an anti-myc immunoprecipitation assay, and identified the Mtw1-HA tagged protein by Western blot. The interaction of Spc25 with Mtw1 was slightly increased or unaffected in the absence of Ybp2 (Figure 5B). Next, we checked whether the lack of Ybp2 disrupted the interaction between Ctf19 in the COMA complex and Mtw1 in the MIND complex. The Ctf19–Mtw1 interaction was slightly increased in the absence of Ybp2 (Figure 5C). Finally, we checked whether the lack of Ybp2 disrupted the interaction between Ctf19 in the COMA complex and Ndc80 in the Ndc80 complex. Unexpectedly, the Ctf19–Ndc80 interaction was increased in the absence of Ybp2 (Figure 5D). Together, these results indicate that the lack of Ybp2 enhances the interactions among the central kinetochore subcomplexes (COMA, MIND, and Ndc80).

Next, we examined the association of the central kinetochore proteins with *CEN* DNA in the absence of Ybp2. The association of Ndc80, Ctf19 and Mcm21 but not Mtw1, Nuf2 or Dsn1 with *CEN* DNA increased in *ybp2Δ* cells (Figures 5E and F), which is consistent with the protein–protein interaction results. Taken together, it appears that Ybp2 may push the central kinetochore subcomplexes away rather than hold them together.

### *YBP2* Genetically Interacts with the Genes that Encode the COMA Components

Because Ybp2 appears to coprecipitate more efficiently with Ctf19 and Mcm21 (Figure 3A) than with other kinetochore proteins and because the *CEN* binding of Ctf19 and Mcm21 was dramatically affected in the absence of Ybp2 (Figures 5E and F), we tested the genetic interaction between *YBP2* and *CTF19* or *MCM21*. All deletion mutants (*ybp2*Δ, *ctf19*Δ, and *mcm21*Δ) grew normally on YPD at 25°C or 37°C, but the *ybp2*Δ*ctf19*Δ and *ybp2*Δ*mcm21*Δ double mutants and the *ybp2*Δ*ctf19*Δ*mcm21*Δ triple mutant failed to grow at 37°C (Figure 5G, top and middle). On the other hand, the *ctf19*Δ*mcm21*Δ double mutant grew normally on YPD at 37°C (Figure 5G, top and middle). Thus, a *ybp2*Δ mutation genetically interacts with mutations in *CTF19* or *MCM21*, which suggests that Ybp2 supports the essential function of the COMA complex. Consistent with these observations, *ybp2Δ* exhibited temperature-sensitive conditional synthetic-lethal interactions with *ame1-4* or *okp1-5*, respectively (Figure 5G, bottom).

## Discussion

In this study, we characterized the mitotic function of Ybp2, which was identified by an SGA screen by using the *mad2*-deletion (*mad2*Δ) strain. *ybp2*Δ mutants displayed phenotypes commonly observed in kinetochore mutants, such as sensitivity to the benomyl, G2/M accumulation when cycling, and chromosome missegregation. Ybp2 associates with several central kinetochore proteins and the centromeric histone Cse4. It also associates with *CEN* DNA in an Ndc10-dependent manner. *YBP2* shows genetic interactions with the kinetochore genes *CTF19* and *MCM21.* These results strongly support the role of Ybp2 in kinetochore function.

### SGA Screens That Use a Spindle Checkpoint Mutant Identified Mitotic Factors

An SGA screen that uses a *mad2*Δ mutant strain identified the 4 novel mitotic genes (YBR194W, YDR359C, YGL060W, YJL064W) [24]. When the screen identified the genes, no gene had been previously characterized. However, while we were characterizing these genes, studies on YDR359C and YGL060W were published by other investigators, who designated these genes as *VID21* [39] and *YBP2/YBH1* [36], respectively. Vid21 is required for bulk histone H4 acetylation and is functionally linked to the histone H2A variant Htz1 and the Swr1 ATPase complex (SWR-C) that assembles Htz1 into chromatin [39]. Ybp2 influences H_2_O_2_ tolerance [36], though its role in this function appears marginal. The functions of the other genes are yet unknown. However, YJL064W is dubious and overlaps with *DLS1*; therefore, *DLS1* may be the real target of Mad2. Genetic interactions between *YBP2* and *MAD2* have also been identified by Daniel et al. [40], thus providing an independent confirmation of our findings.

Importantly, none of these genes, including *YBP2*, has been identified as a mitotic factor by previous proteomic methods. Thus, genetic screens complement proteomic approaches and hold promise for identifying novel mitotic factors that are *functionally* related to known mitotic factors.

### Kinetochore Function of Ybp2

Based on protein–protein and protein–DNA coimmunoprecipitation results, we describe a Ybp2 network of physical interactions in the kinetochore. Ybp2 interacts with proteins in the COMA and Ndc80 complexes, but not substantially with those in the MIND complex. We hypothesized that the 3 complexes have a 3-dimensional surface by assuming that Ybp2 is a structural component of the central kinetochore. However, coimmunoprecipitation data generated by using the *ybp2*Δ mutant suggest that lack of Ybp2 enhances the interactions among the central kinetochore subcomplexes. ChIP assays showed that *CEN* binding of Ctf19, Mcm21, and Ndc80 also increased in *ybp2Δ* cells. Interestingly, Ybp2 localizes throughout the cell (Supplemental Figure S7, [36]). One possibility is that Ybp2 may not be merely a structural component of the kinetochore complex, but have a chaperone function by helping arrange the central kinetochore subcomplexes.

When the spindle checkpoint is active, loss of Ybp2 does not cause a substantial mitotic defect but does cause a mitotic delay. When the spindle checkpoint is inactive, the loss of Ybp2 results in substantial chromosome loss, suggesting that the spindle checkpoint detects the mitotic defect and suppresses it by delaying mitosis.

We also observed a synthetic sickness phenotype for *ybp2*Δ*ctf19*Δ and *ybp2*Δ*mcm21*Δ double mutants. Apart from Okp1 and Ame1, Ctf19 and Mcm21 are nonessential in the COMA complex. Because we did not see any growth defect in the *ctf19*Δ*mcm21*Δ double mutant, Ybp2 may support the essential function of the COMA complex more specifically than it supports that of the other central kinetochore complexes.

The *ybp2*Δ mutant arrested with G2/M DNA content in haploid cells containing a nonessential marked chromosome fragment and in normal diploid cells, but not in normal haploid cells. This interesting finding suggests that there is some specific mechanistic difference between the mitotic chromosome segregation of aneuploid haploids and normal diploid cells, but further investigation is required to address this issue.

### Ybp2 Shares Domains with Slk19

Ybp2 is highly conserved within fungi. Hpc2 (26% identity and 46% similarity) in *Candida albicans* and Cagl0k06743g (32% identity and 52% similarity) and Cagl0f06985g (30% identity and 49% similarity) in *Candida glabrata* are putative Ybp2 homologs (the Yeast Protein Database; https://www.proteome.com/tools/proteome/databases.jsp).

Ybp2 has 3 sequence blocks that are homologous to the *S. cerevisiae* kinetochore protein Slk19 (Supplemental Figure S8A). These blocks are also conserved in the human homologue of Slk19, CCDC73 (unpublished data). Slk19 is important for proper chromosome segregation and is found at kinetochores in metaphase and at the spindle midzone in anaphase [41]. Slk19 was identified as a component of the FEAR (Cdc Fourteen Early Anaphase Release) network, which promotes Cdc14 release from the nucleolus during early anaphase [42]. We found that the *ybp2*Δ mutation genetically interacts with the *slk19*Δ mutation (Supplemental Figure S8B). Therefore, Ybp2 may be functionally related to Slk19.

### *YBP2* is a Polymorphic Gene

We have found polymorphisms in *YBP2*. A sequence comparison of *YBP2* of BY4741 (SGA background) and YPH499 (our background) has revealed 7 different bases in the open reading frame (1926 bp), of which 3 affect the amino acid sequences (Supplemental Figure S9). We have not found any functional relevance of these polymorphisms yet.

## Materials and Methods

### Yeast strains and medium

Supplemental Table S1 presents the genotypes of yeast strains used for this study. The medium for yeast growth and sporulation has been previously described [43]. Yeast strains were transformed by the method of Ito and coworkers [44]. Strains that expressed myc-tagged proteins were generated by the procedure of Longtine and coworkers [45]. For the microtubule-depolymerizing drug sensitivity assay, benomyl (DuPont, Wilmington, DE) was added at the indicated concentration to the YPD medium; only dimethyl sulfoxide was added to the YPD medium as a control.

### Colony color assay

The colony color assay was performed as previously described [33], [46]. In brief, each strain containing a single *SUP11*-marked chromosome fragment was plated at a density of ∼200 colonies per plate on minimal (SD) medium containing a limiting amount of adenine (6 µg/mL) and grown at 30°C. A colony consists of cells containing the chromosome fragment (white) and cells that have lost it (red), resulting in a white-and-red sectored phenotype.

### Antibodies

Anti-Ybp2 antibodies were generated as previously described [47]–[49]. Anti-myc (Roche, Indianapolis, IN), antihemagglutinin (anti-HA; Roche, Indianapolis, IN), and antitubulin (Serotech, Oxford, UK) antibodies were purchased.

### ChIP assay and coimmunoprecipitation analyses

Immunoprecipitation using yeast lysates was performed as described previously [47]. ChIP assays of yeast lysates were performed by a previously described method [20], with the following modifications. The primer pairs used to amplify specific regions of DNA are detailed by Meluh and Koshland [50]. The expected sizes of PCR products were 302 bp (*CEN1*), 344 bp (*CEN16*), and 288 bp (*PGK1*). The total chromatin added varied from 1/1500 to 1/600 and the immunoprecipitated chromatin template varied from 1/10 to 1/30 of the available template, depending on the linear range for PCR. Band intensities of X-ray films and photographs were quantified with the Quantity One (Version 4.6.2) software. Binding activity was defined as the ratio of the amount of coprecipitated protein or DNA to the amount of input protein or DNA.

### Immunofluorescence

Indirect immunofluorescent staining were performed as described previously [51], with the following modifications. Yeast cells were grown to log phase and fixed with 3.7% formaldehyde for 15 min at 30°C. Cells were collected by centrifugation, washed twice with SK (1 M sorbitol and 50 mM KH_2_PO_4_, pH 7.5), and resuspended in 1 mL of SK plus 0.01% 2-mercaptoethanol and zymolyase (final concentration 34 µg/mL). After digestion for 15 min at 37°C, cells were washed twice with SK and applied to polylysine-coated multiwell slides. Cells were washed twice with SK, blocked in 10% bovine serum albumin (BSA)-PBS for 20 min at room temperature, and incubated with the primary antibody for 60 min at 37°C. The primary antibody was diluted 1∶50 (rat antitubulin) or 1∶200 (mouse anti-myc) in 10% BSA-PBS. Cells were washed 7 times with PBS and incubated with the fluorescent secondary antibody for 90 min at 37°C. The secondary antibody, fluorescein isothiocyanate (FITC)-conjugated AffiniPure goat anti-rat IgG (Jackson ImmunoResearch Laboratories, West Grove, PA) or fluorescein isothiocyanate (FITC)-conjugated AffiniPure goat anti-mouse IgG (Jackson ImmunoResearch Laboratories, West Grove, PA), was diluted to 1∶1000 in 10% BSA-PBS. Cells were washed 7 times with PBS and incubated in 4′,6-diamidino-2-phenylindole (DAPI; 100 ng/mL). After washing several times with PBS, cells were observed through a Leica DM IRE2 motorized fluorescence microscope equipped with an HCX PL APO 100× oil immersion lens (Leica, Wetzlar, Germany), an ARC LAMP power supply HBO100 DC IGN (Ludl Electronic Products, Hawthorne, NY), and an ORCA-ER high-resolution digital CCD camera (Hamamatsu, Hamamatsu City, Japan). Image acquisition and processing were performed with the Openlab version 4 Scientific Imaging Software (Improvision, Lexington, MA).

## Supporting Information

Figure S1 The *ybp2*Δ/*ybp2*Δ cells accumulate at the G2/M phase of the cell cycle. Logarithmically growing cells, wild type (YPH501) and *ybp2*Δ*/ybp2*Δ (Y1847), were cultured at 25 °C and processed for flow cytometry.
(0.15 MB TIF)

Figure S2 The *ybp2*Δ mutant does not exhibit an endogenous chromosome III missegregation phenotype. A faker method (top panel) or a diploid bimater method (bottom panel) [52] was used. *Mat*α *ybp2*Δ strain (Y1335) was mated with α tester strain (17/17); the *ybp2*Δ*/ybp2*Δ diploid strain (Y1847) was mated with haploid-tester strains (a tester: 17/14, α tester: 17/17); and mating products were selected.
(2.33 MB TIF)

Figure S3 Ybp2 is not required for the spindle checkpoint. (A) Logarithmically growing cells were treated with 15 µg/mL nocodazole for 100 min at 30 °C. Samples were taken both before (-Nocodazole) and after treatment (+Nocodazole), fixed, stained for DNA, and analyzed by flow cytometry. Isogenic yeast strains were wild type (Y14), *ybp2*Δ (Y1831), *mad2*Δ (Y1833). (B) The spindle checkpoint was activated in *ctf8*Δ*ybp2*Δ as well as *ctf8*Δ cells. Wild-type (Y863), *ctf8*Δ (Y899), and *ctf8*Δ*ybp2*Δ (Y1858) cells were arrested in G1 with 5 µg/mL α-factor and released into the YPD medium. Samples were taken at the indicated time point. Lysates were prepared and immunoblotted with anti-myc antibody to analyze the Pds1 protein level. Equal protein concentrations were loaded in all lanes, as judged by the Cdc28 protein level. *ctf8*Δ*ybp2*Δ cells activated the spindle checkpoint as well as *ctf8*Δ cells.
(1.89 MB TIF)

Figure S4 Ybp2 is not required for sister chromatid cohesion. The sister chromatid cohesion assay was performed by a previously described method [30]. Wild-type (SBY818) and *ybp2*Δ (Y1859) cells were arrested in G1 with 5 µg/mL α-factor or in G2/M with 15 µg/mL nocodazole. To visualize sister chromatids, copper sulfate was added to the medium at a final concentration of 0.25 µM to induce the GFP-lacI fusion protein, which is under the control of the copper promoter. For each sample, 100 cells were counted.
(0.15 MB TIF)

Figure S5 Ybp2 does not coimmunoprecipitated with Slk19, Mif2, Ndc10 or Ipl1. The indicated strains were applied for immunoprecipitations as described in Figure 3. Slk19-myc (Y1720), Mif2-myc (Y1705), Ndc10-myc (YVM731A), and Ipl1-myc (Y1723).
(0.75 MB TIF)

Figure S6 Quantification of coimmunoprecipitated *CEN1* and *CEN16* signals of Ybp2-myc and comparison with those of Okp1-myc. Quantification was performed as described in Figure 4.
(0.21 MB TIF)

Figure S7 Ybp2 localizes everywhere in cells. Immunofluorescence analysis of untagged (YPH499) and myc-tagged Ybp2 (Y1689) cells fixed and stained with anti-myc antibodies and DAPI.
(1.09 MB TIF)

Figure S8 Ybp2 is a potential family of Slk19. (A) Comparison of amino acid sequences of Ybp2 and Slk19. Three conserved sequence blocks are shown in black. Black boxes are identical amino acids; gray boxes are similar amino acids. (B) *ybp2*Δ mutations genetically interact with *slk19*Δ mutations. Yeast strains were spotted in 5-fold dilutions from 5×10^4^ cells per spot on YPD plates. The plates were incubated at the indicated temperatures for 2 days. Isogenic yeast strains used were wild type (YPH499), *ybp2*Δ (Y1337), *slk19*Δ (Y1860), and *ybp2*Δ*slk19*Δ (Y1861).
(1.21 MB TIF)

Figure S9 Ybp2 polymorphism. Ybp2 consists of 641 amino acids. The amino acid sequence of Ybp2 in BY4741 corresponds with that in the *Saccharomyces* Genome Database. Asterisks indicate the positions at which sequences are different between Ybp2 of BY4741 and YPH499. Amino acids are shown in linear boxes.
(0.14 MB TIF)

Table S1 Yeast strains used in this study.
(0.18 MB DOC)
