# Supplementary figures and images for: Ybp2 Associates with the Central Kinetochore of Saccharomyces cerevisiae and Mediates Proper Mitotic Progression
Source: PLoS One. 2008 Feb 20;3(2):e1617. doi: 10.1371/journal.pone.0001617 (PMC2238814; doi:10.1371/journal.pone.0001617)

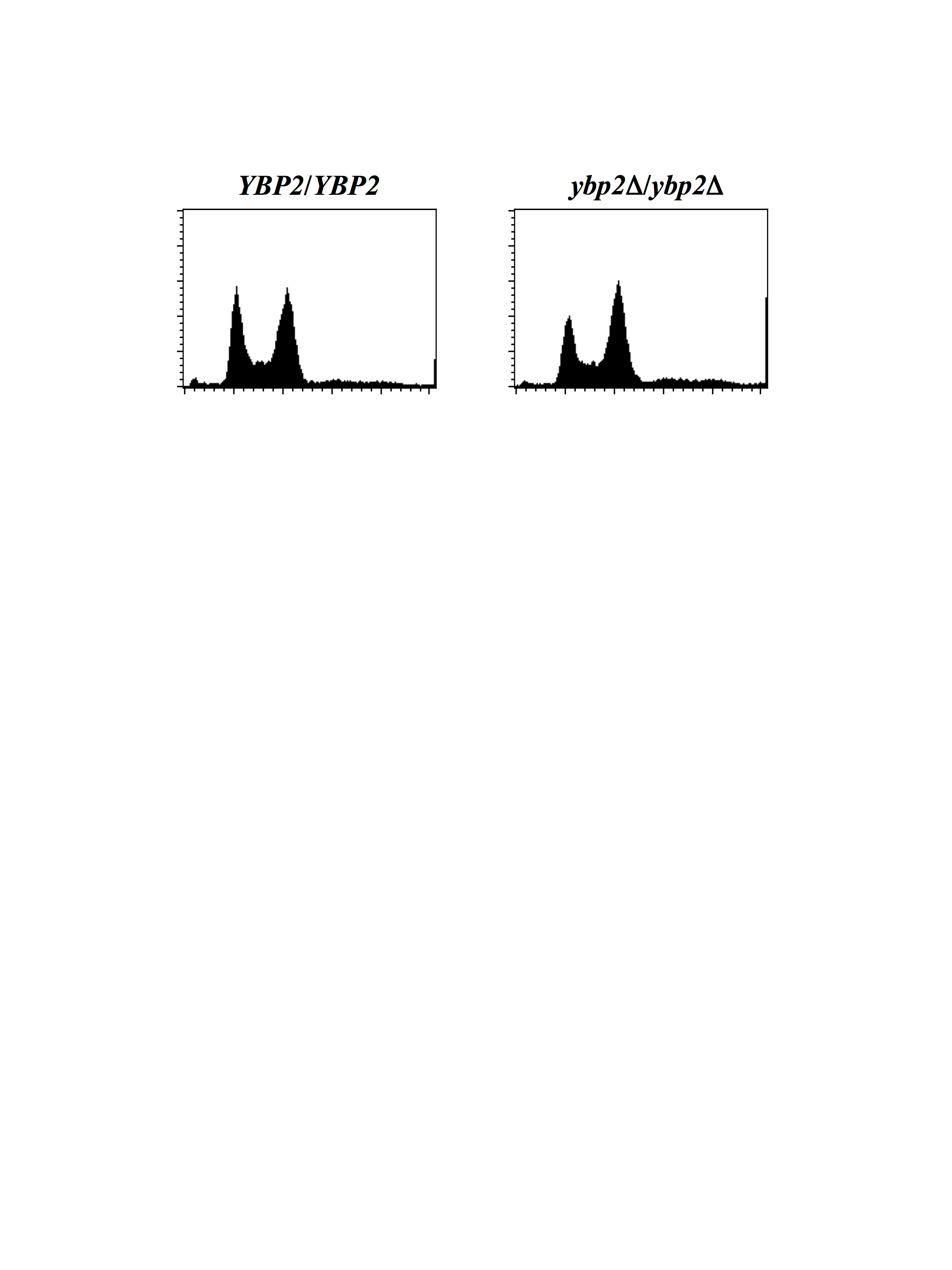

Supplement: Figure S1 — The ybp2Δ/ybp2Δ cells accumulate at the G2/M phase of the cell cycle. Logarithmically growing cells, wild type (YPH501) and ybp2Δ/ybp2Δ (Y1847), were cultured at 25 °C and processed for flow cytometry. (0.15 MB TIF) [file pone.0001617.s001.tif]

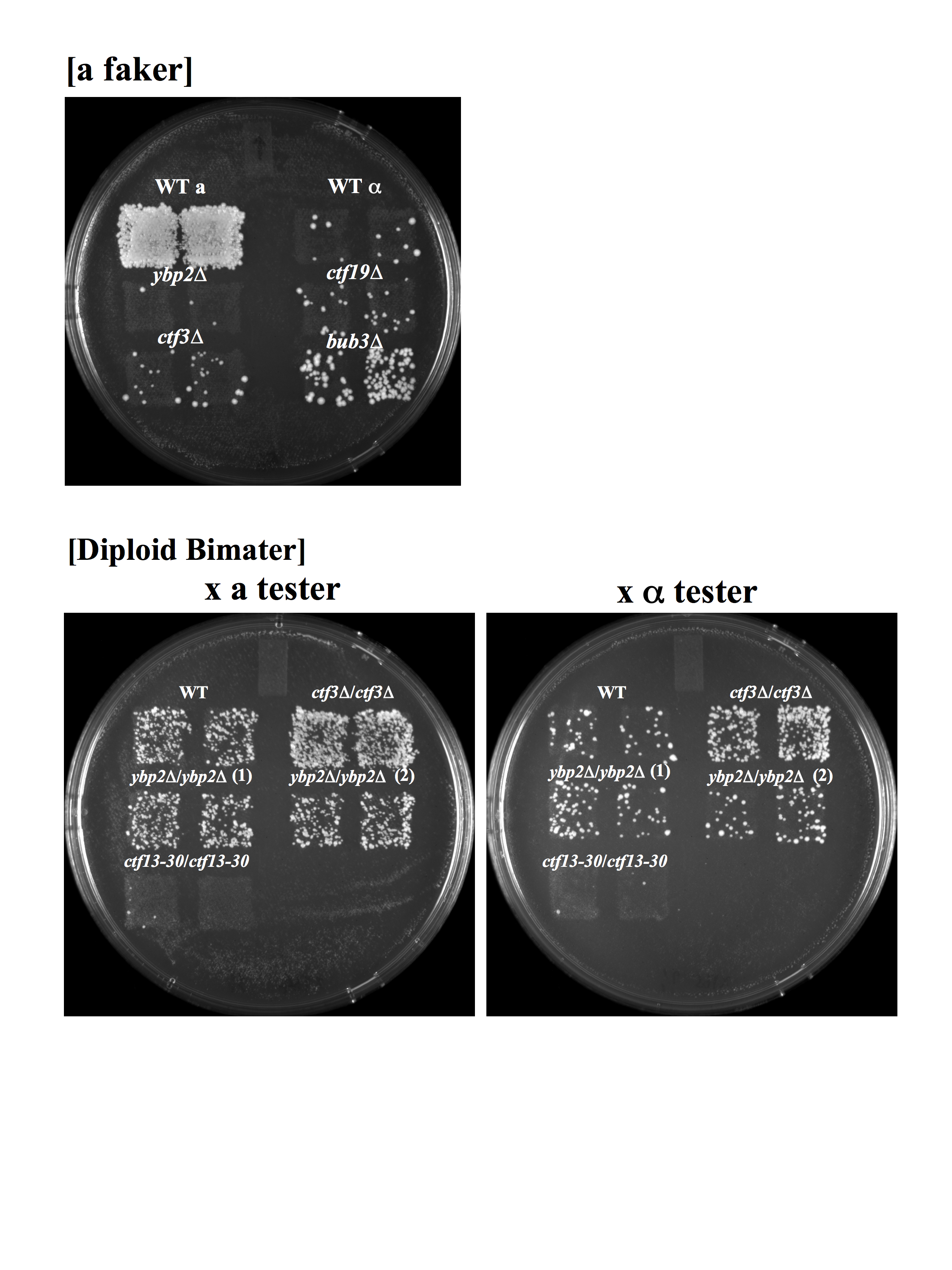

Supplement: Figure S2 — The ybp2Δ mutant does not exhibit an endogenous chromosome III missegregation phenotype. A faker method (top panel) or a diploid bimater method (bottom panel) [52] was used. Matα ybp2Δ strain (Y1335) was mated with α tester strain (17/17); the ybp2Δ/ybp2Δ diploid strain (Y1847) was mated with haploid-tester strains (a tester: 17/14, α tester: 17/17); and mating products were selected. (2.33 MB TIF) [file pone.0001617.s002.tif]

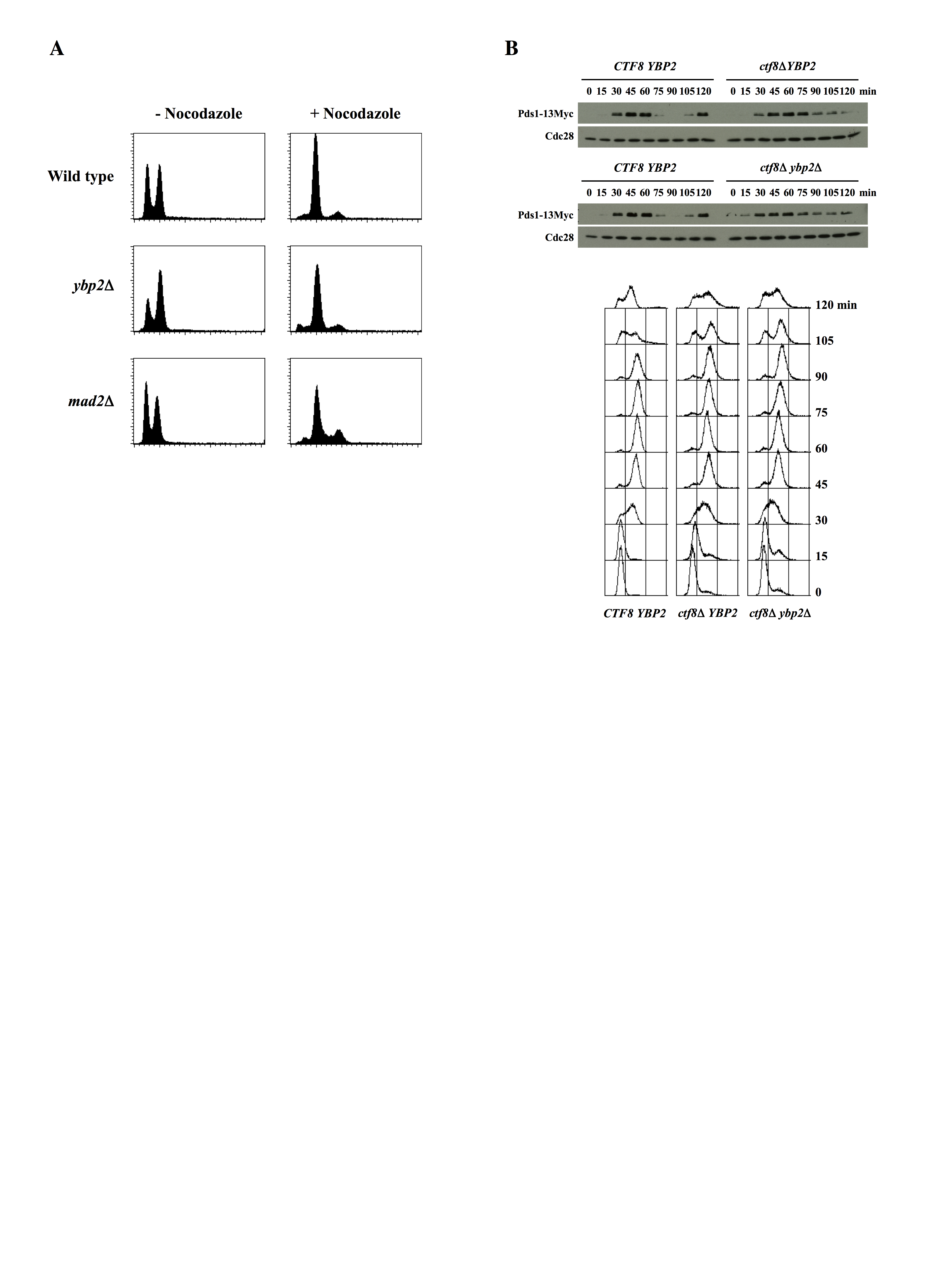

Supplement: Figure S3 — Ybp2 is not required for the spindle checkpoint. (A) Logarithmically growing cells were treated with 15 µg/mL nocodazole for 100 min at 30 °C. Samples were taken both before (-Nocodazole) and after treatment (+Nocodazole), fixed, stained for DNA, and analyzed by flow cytometry. Isogenic yeast strains were wild type (Y14), ybp2Δ (Y1831), mad2Δ (Y1833). (B) The spindle checkpoint was activated in ctf8Δybp2Δ as well as ctf8Δ cells. Wild-type (Y863), ctf8Δ (Y899), and ctf8Δybp2Δ (Y1858) cells were arrested in G1 with 5 µg/mL α-factor and released into the YPD medium. Samples were taken at the indicated time point. Lysates were prepared and immunoblotted with anti-myc antibody to analyze the Pds1 protein level. Equal protein concentrations were loaded in all lanes, as judged by the Cdc28 protein level. ctf8Δybp2Δ cells activated the spindle checkpoint as well as ctf8Δ cells. (1.89 MB TIF) [file pone.0001617.s003.tif]

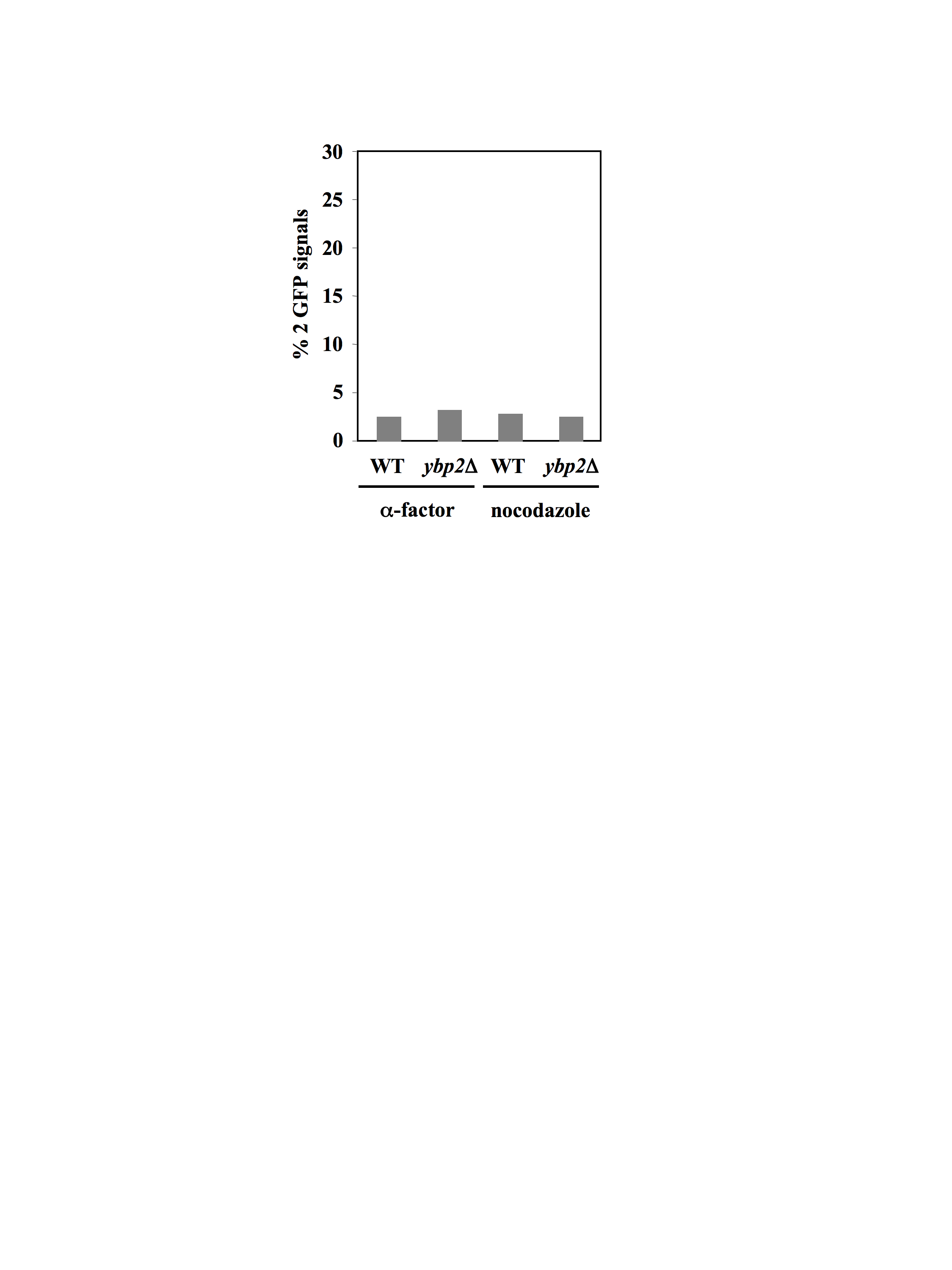

Supplement: Figure S4 — Ybp2 is not required for sister chromatid cohesion. The sister chromatid cohesion assay was performed by a previously described method [30]. Wild-type (SBY818) and ybp2Δ (Y1859) cells were arrested in G1 with 5 µg/mL α-factor or in G2/M with 15 µg/mL nocodazole. To visualize sister chromatids, copper sulfate was added to the medium at a final concentration of 0.25 µM to induce the GFP-lacI fusion protein, which is under the control of the copper promoter. For each sample, 100 cells were counted. (0.15 MB TIF) [file pone.0001617.s004.tif]

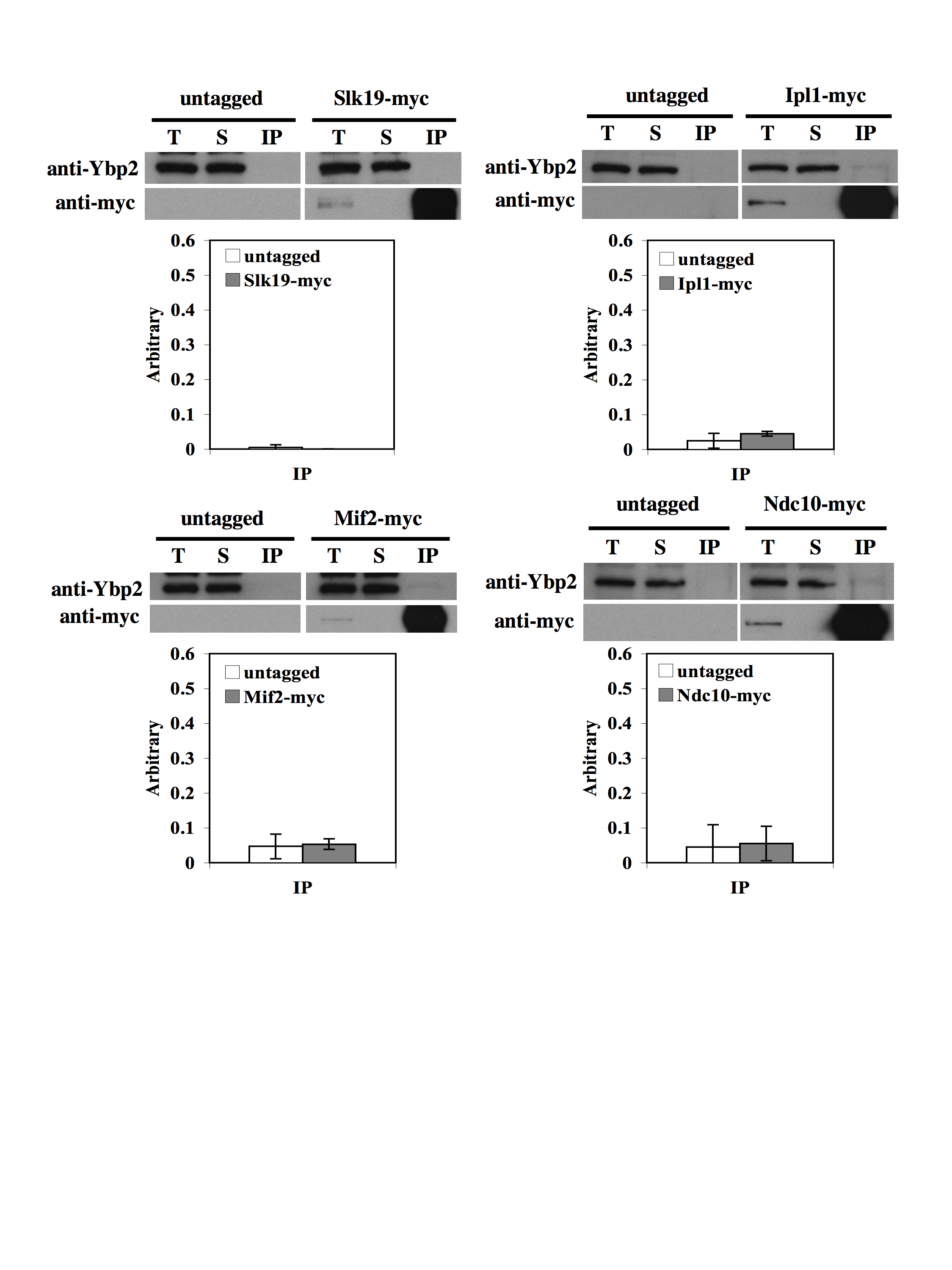

Supplement: Figure S5 — Ybp2 does not coimmunoprecipitated with Slk19, Mif2, Ndc10 or Ipl1. The indicated strains were applied for immunoprecipitations as described in Figure 3. Slk19-myc (Y1720), Mif2-myc (Y1705), Ndc10-myc (YVM731A), and Ipl1-myc (Y1723). (0.75 MB TIF) [file pone.0001617.s005.tif]

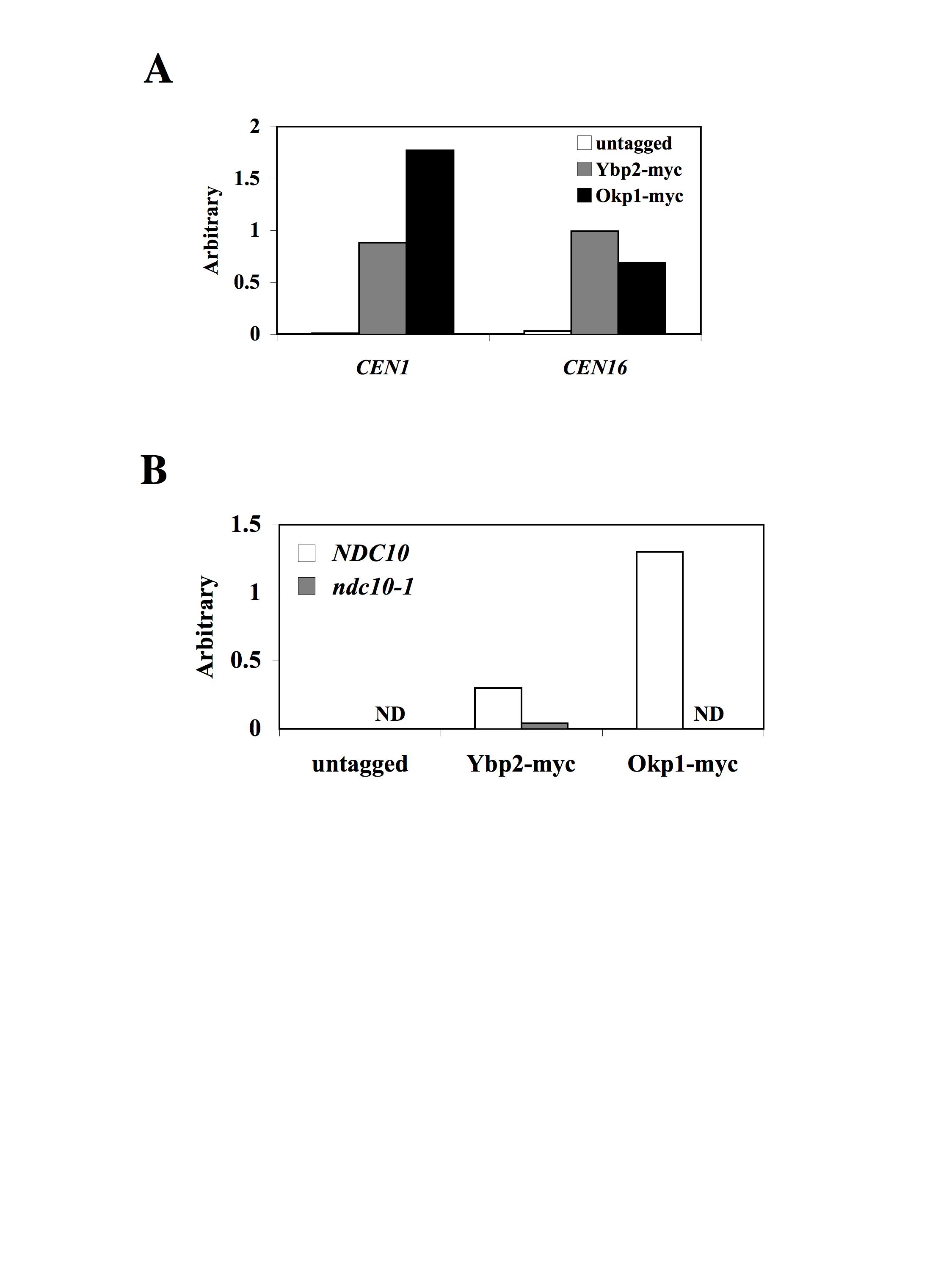

Supplement: Figure S6 — Quantification of coimmunoprecipitated CEN1 and CEN16 signals of Ybp2-myc and comparison with those of Okp1-myc. Quantification was performed as described in Figure 4. (0.21 MB TIF) [file pone.0001617.s006.tif]

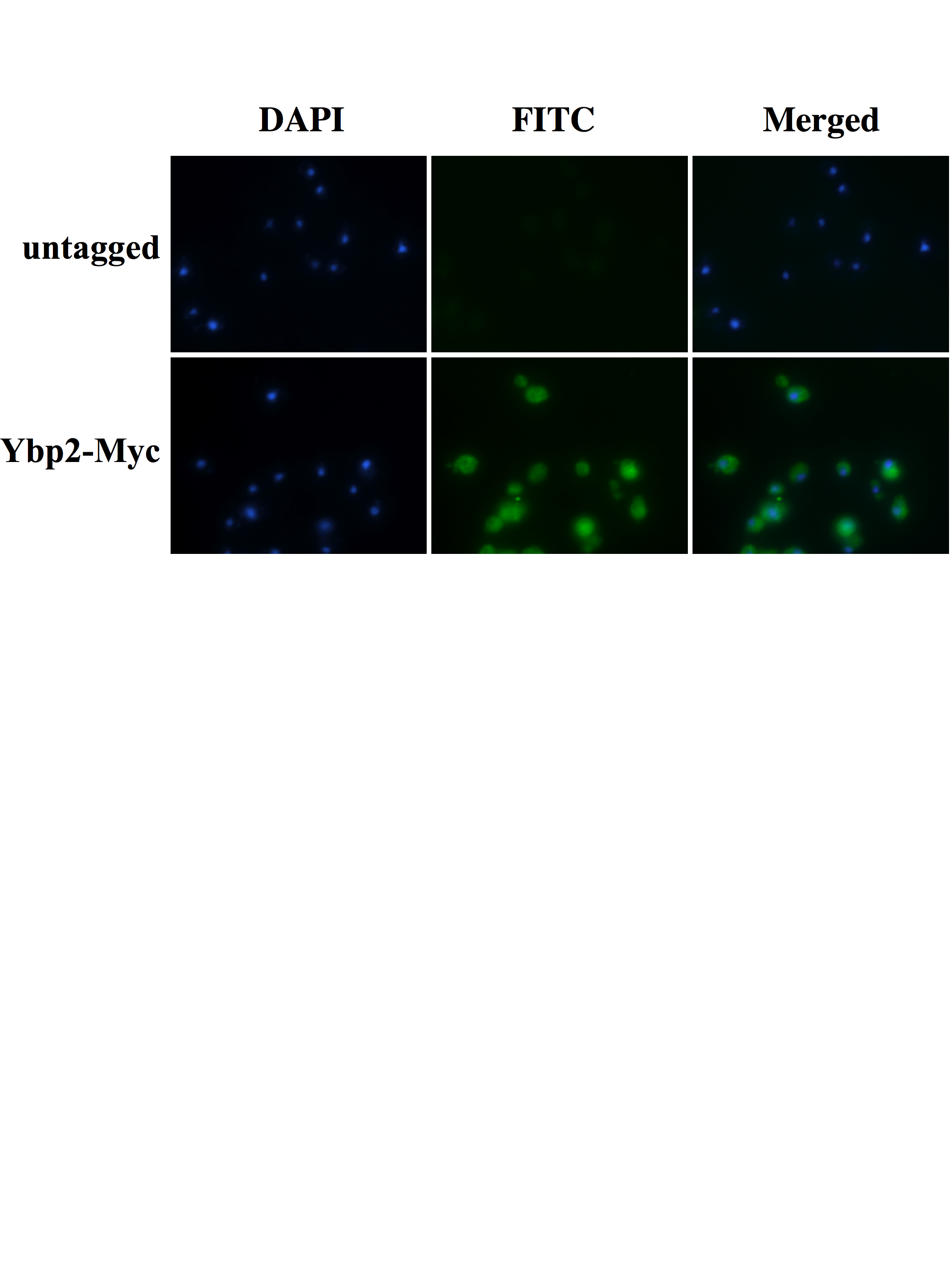

Supplement: Figure S7 — Ybp2 localizes everywhere in cells. Immunofluorescence analysis of untagged (YPH499) and myc-tagged Ybp2 (Y1689) cells fixed and stained with anti-myc antibodies and DAPI. (1.09 MB TIF) [file pone.0001617.s007.tif]

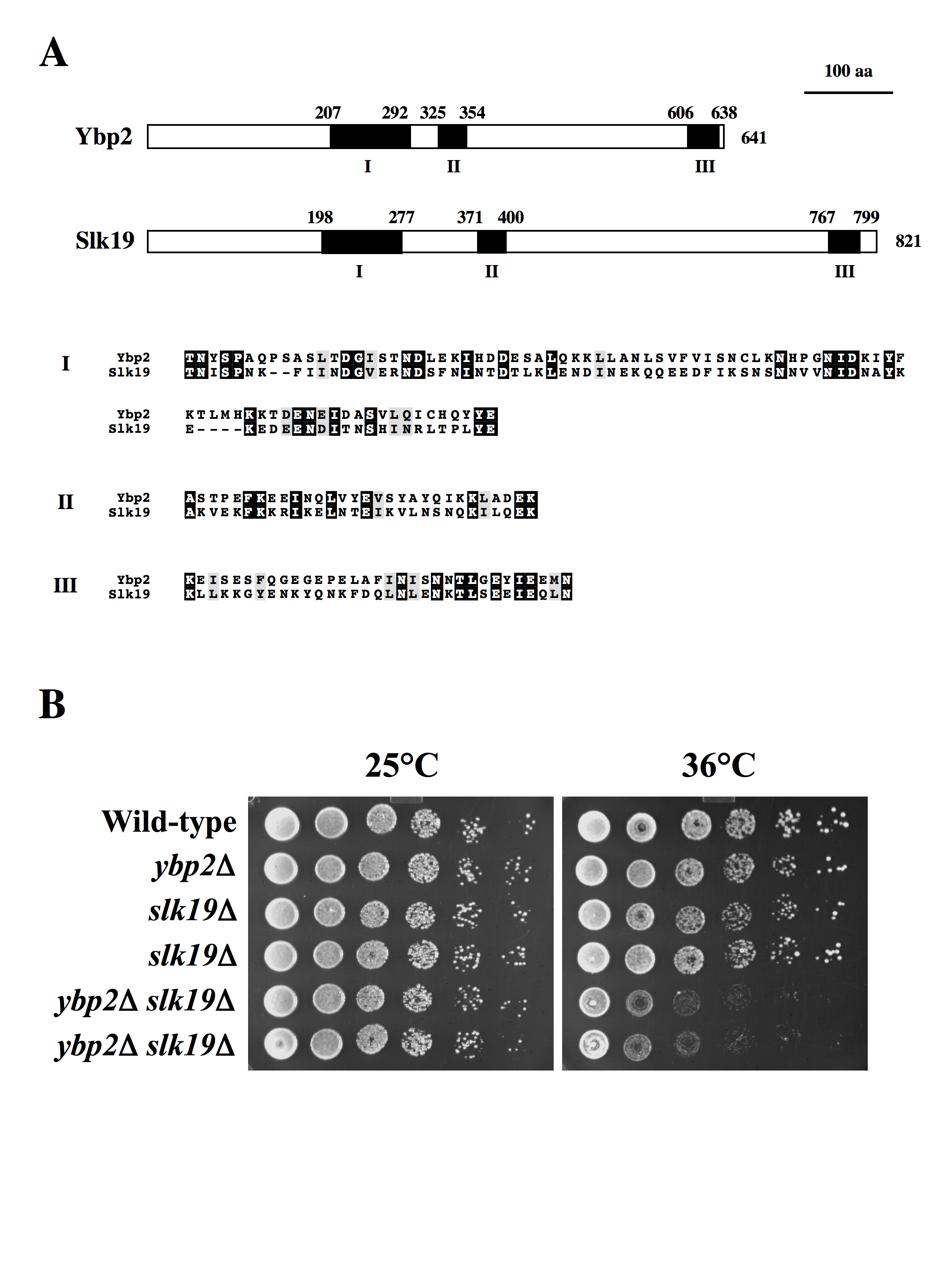

Supplement: Figure S8 — Ybp2 is a potential family of Slk19. (A) Comparison of amino acid sequences of Ybp2 and Slk19. Three conserved sequence blocks are shown in black. Black boxes are identical amino acids; gray boxes are similar amino acids. (B) ybp2Δ mutations genetically interact with slk19Δ mutations. Yeast strains were spotted in 5-fold dilutions from 5×10^4^ cells per spot on YPD plates. The plates were incubated at the indicated temperatures for 2 days. Isogenic yeast strains used were wild type (YPH499), ybp2Δ (Y1337), slk19Δ (Y1860), and ybp2Δslk19Δ (Y1861). (1.21 MB TIF) [file pone.0001617.s008.tif]

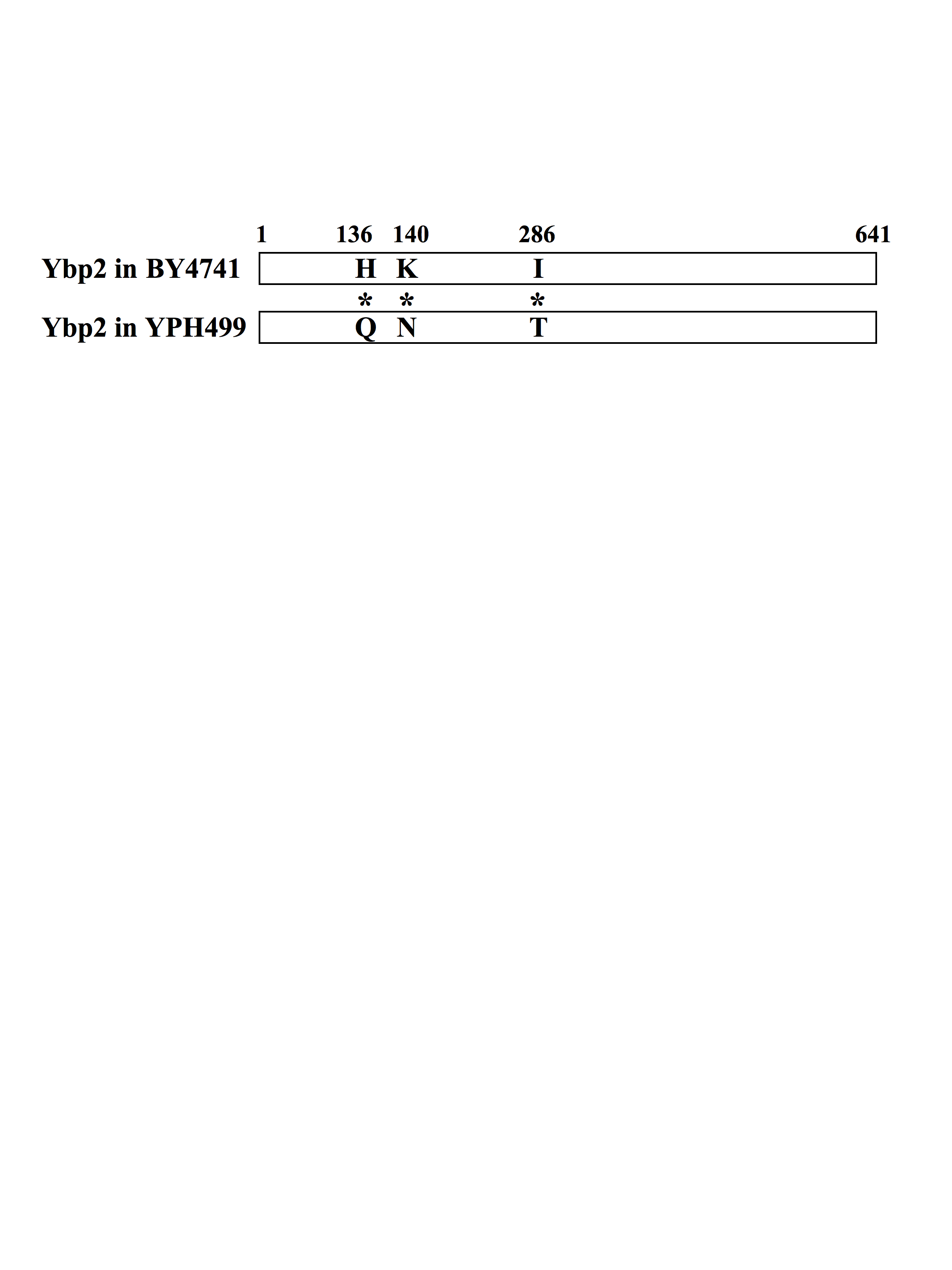

Supplement: Figure S9 — Ybp2 polymorphism. Ybp2 consists of 641 amino acids. The amino acid sequence of Ybp2 in BY4741 corresponds with that in the Saccharomyces Genome Database. Asterisks indicate the positions at which sequences are different between Ybp2 of BY4741 and YPH499. Amino acids are shown in linear boxes. (0.14 MB TIF) [file pone.0001617.s009.tif]
