# Supplementary material for: Ybp2 Associates with the Central Kinetochore of Saccharomyces cerevisiae and Mediates Proper Mitotic Progression
Source: PLoS One. 2008 Feb 20;3(2):e1617. doi: 10.1371/journal.pone.0001617 (PMC2238814; doi:10.1371/journal.pone.0001617)
Supplement: Table S1 — Yeast strains used in this study. (0.18 MB DOC) [file pone.0001617.s010.doc]

| Strain | Genotype | Reference |
| --- | --- | --- |
| BY4741 | *MAT***a** *his3*∆*1* *leu2*∆*0* *met15*∆*0 ura3*∆*0* | Open Biosystems |
| Y516 | *MAT*α *his3*∆*1* *leu2*∆*0* *lys2*∆*0 ura3*∆*0 mad2*∆*::kanMX6* | Research Genetic |
| Y1028 | *MAT***a** *his3*∆*1* *leu2*∆*0* *met15*∆*0 ura3*∆*0 ybp2*∆*::kanMX6* | Open Biosystems |
| YPH499 | *MAT***a** *ura3-52 lys2-801 ade2-101 trp1*∆*63 his3*∆*200 leu2*∆*1* | [53] |
| YPH500 | *MAT*α *ura3-52 lys2-801 ade2-101 trp1*∆*63 his3*∆*200 leu2*∆*1* | [53] |
| Y530 | *MAT*α *ura3-52 lys2-801 ade2-101 trp1*∆*63 his3*∆*200 leu2*∆*1 bub1*∆*::LEU2* | This study |
| Y548 | *MAT*α *ura3-52 lys2-801 ade2-101 trp1*∆*63 his3*∆*200 leu2*∆*1 bub3*∆*::kanMX6* | [48] |
| Y550 | *MAT*α *ura3-52 lys2-801 ade2-101 trp1*∆*63 his3*∆*200 leu2*∆*1 mad1*∆*::kanMX6* | [48] |
| Y554 | *MAT*α *ura3-52 lys2-801 ade2-101 trp1*∆*63 his3*∆*200 leu2*∆*1 mad3*∆*::kanMX6* | [48] |
| YPH1315 | *MAT***a** *ura3-52 lys2-801 ade2-101 trp1*∆*63 his3*∆*200 leu2*∆*1 ctf19*∆*::HIS3* | [29] |
| Y1323 | *MAT*α *ura3-52 lys2-801 ade2-101 trp1*∆*63 his3*∆*200 leu2*∆*1 mad2*∆*::TRP1* | This study |
| Y1335 | *MAT*α *ura3-52 lys2-801 ade2-101 trp1*∆*63 his3*∆*200 leu2*∆*1 ybp2*∆*::kanMX6* | This study |
| Y1337 | *MAT***a** *ura3-52 lys2-801 ade2-101 trp1*∆*63 his3*∆*200 leu2*∆*1 ybp2*∆*::kanMX6* | This study |
| Y1342 | *MAT*α *ura3-52 lys2-801 ade2-101 trp1*∆*63 his3*∆*200 leu2*∆*1 ybp2*∆*::His3MX6* | This study |
| Y1824 | *MAT***a** *ura3-52 lys2-801 ade2-101 trp1*∆*63 his3*∆*200 leu2*∆*1 mcm21*∆*::MX4-natR* | This study |
| Y1860 | *MAT***a** *ura3-52 lys2-801 ade2-101 trp1*∆*63 his3*∆*200 leu2*∆*1 slk19*∆*::His3MX6* | This study |
| YPH1676 | *MAT***a** *ura3-52 lys2-801 ade2-101 trp1*∆*63 his3*∆*200 leu2*∆*1 ame1-4:TRP1* | [54] |
| YPH1678 | *MAT***a** *ura3-52 lys2-801 ade2-101 trp1*∆*63 his3*∆*200 leu2*∆*1 okp1-5:TRP1* | [54] |
| Y1396 | *MAT*α *ura3-52 lys2-801 ade2-101 trp1*∆*63 his3*∆*200 leu2*∆*1 ybp2*∆*::His3MX6 bub3*∆*::kanMX6* | This study |
| Y1398 | *MAT*α *ura3-52 lys2-801 ade2-101 trp1*∆*63 his3*∆*200 leu2*∆*1 ybp2*∆*::His3MX6 mad1*∆*::kanMX6* | This study |
| Y1402 | *MAT*α *ura3-52 lys2-801 ade2-101 trp1*∆*63 his3*∆*200 leu2*∆*1 ybp2*∆*::His3MX6 mad3*∆*::kanMX6* | This study |
| Y1415 | *MAT*α *ura3-52 lys2-801 ade2-101 trp1*∆*63 his3*∆*200 leu2*∆*1 ybp2*∆*::His3MX6 bub1*∆*::LEU2* | This study |
| Y1419 | *MAT*α *ura3-52 lys2-801 ade2-101 trp1*∆*63 his3*∆*200 leu2*∆*1 ybp2*∆*::His3MX6 mad2*∆*::TRP1* | This study |
| Y1826 | *MAT***a** *ura3-52 lys2-801 ade2-101 trp1*∆*63 his3*∆*200 leu2*∆*1 ybp2*∆*::kanMX6 ctf19*∆*::MX4-natR* | This study |
| Y1827 | *MAT***a** *ura3-52 lys2-801 ade2-101 trp1*∆*63 his3*∆*200 leu2*∆*1 ybp2*∆*::kanMX6 mcm21*∆*::MX4-natR* | This study |
| Y1828 | *MAT***a** *ura3-52 lys2-801 ade2-101 trp1*∆*63 his3*∆*200 leu2*∆*1 ctf19*∆*::HIS3 mcm21*∆*::MX4-natR* | This study |
| Y1861 | *MAT***a** *ura3-52 lys2-801 ade2-101 trp1*∆*63 his3*∆*200 leu2*∆*1 ybp2∆::KanMX6 slk19*∆*::His3MX6* | This study |
| Y1864 | *MAT***a** *ura3-52 lys2-801 ade2-101 trp1*∆*63 his3*∆*200 leu2*∆*1 ybp2∆::His3MX6 ame1-4:TRP1* | This study |
| Y1865 | *MAT***a** *ura3-52 lys2-801 ade2-101 trp1*∆*63 his3*∆*200 leu2*∆*1 ybp2∆::HisMX6 okp1-5:TRP1* | This study |
| Y1829 | *MAT***a** *ura3-52 lys2-801 ade2-101 trp1*∆*63 his3*∆*200 leu2*∆*1 ybp2*∆*::kanMX6 ctf19*∆*::HIS3 mcm21*∆*::MX4-natR* | This study |
| Y14 | *MAT***a** *ura3-52 lys2-801 ade2-101 trp1*∆*63 his3*∆*200 leu2*∆*1* CFIII (*CEN3.L.*YPH983) *TRP1 SUP11* | P. Hieter |
| Y1831 | *MAT***a** *ura3-52 lys2-801 ade2-101 trp1*∆*63 his3*∆*200 leu2*∆*1* CFIII (*CEN3.L.*YPH983) *TRP1 SUP11 ybp2*∆*::kanMX6* | This study |
| Y1833 | *MAT***a** *ura3-52 lys2-801 ade2-101 trp1*∆*63 his3*∆*200 leu2*∆*1* CFIII (*CEN3.L.*YPH983) *TRP1 SUP11 mad2*∆*::His3MX6* | This study |
| Y1834 | *MAT***a** *ura3-52 lys2-801 ade2-101 trp1*∆*63 his3*∆*200 leu2*∆*1* CFIII (*CEN3.L.*YPH983) *TRP1 SUP11 ybp2*∆*::kanMX6 mad2*∆*::His3MX6* | This study |
| Y1835 | *MAT***a** *ura3-52 lys2-801 ade2-101 trp1*∆*63 his3*∆*200 leu2*∆*1* CFIII (*CEN3.L.*YPH983) *TRP1 SUP11 ybp1*∆*::MX4-natR* | This study |
| Y1836 | *MAT***a** *ura3-52 lys2-801 ade2-101 trp1*∆*63 his3*∆*200 leu2*∆*1* CFIII (*CEN3.L.*YPH983) *TRP1 SUP11 ybp2*∆*::kanMX6 ybp1*∆*::MX4-natR* | This study |
| Y863 | *MAT***a** *ura3-52 lys2-801 ade2-101 trp1*∆*63 his3*∆*200 leu2*∆*1 PDS1-13Myc:His3MX6* | This study |
| YVM731A | *MAT*α *ura3-52 lys2-801 ade2-101 trp1*∆*63 his3*∆*200 leu2*∆*1 NDC10-13Myc:kanMX6* | V. Measday |
| YPH1542 | *MAT***a** *ura3-52 lys2-801 ade2-101 trp1*∆*63 his3*∆*200 leu2*∆*1 CHL4-13Myc:TRP1* | [23] |
| IPY313 | *MAT***a** *ura3-52 lys2-801 ade2-101 trp1*∆*63 his3*∆*200 leu2*∆*1 CTF19-13Myc:kanMX6* | [20] |
| YVM290 | *MAT***a** *ura3-52 lys2-801 ade2-101 trp1*∆*63 his3*∆*200 leu2*∆*1 MCM22-13Myc:kanMX6* | [20] |
| YVM325 | *MAT***a** *ura3-52 lys2-801 ade2-101 trp1*∆*63 his3*∆*200 leu2*∆*1 MCM16-13Myc:His3MX6* | [20] |
| YVM219 | *MAT***a** *ura3-52 lys2-801 ade2-101 trp1*∆*63 his3*∆*200 leu2*∆*1 CTF3-13Myc:TRP1* | V. Measday |
| Y1689 | *MAT***a** *ura3-52 lys2-801 ade2-101 trp1*∆*63 his3*∆*200 leu2*∆*1 YBP2-13Myc:kanMX6* | This study |
| Y1705 | *MAT***a** *ura3-52 lys2-801 ade2-101 trp1*∆*63 his3*∆*200 leu2*∆*1 MIF2-13Myc:kanMX6* | This study |
| Y1706 | *MAT***a** *ura3-52 lys2-801 ade2-101 trp1*∆*63 his3*∆*200 leu2*∆*1 AME1-13Myc:kanMX6* | This study |
| Y1707 | *MAT***a** *ura3-52 lys2-801 ade2-101 trp1*∆*63 his3*∆*200 leu2*∆*1 OKP1-13Myc:kanMX6* | This study |
| Y1708 | *MAT***a** *ura3-52 lys2-801 ade2-101 trp1*∆*63 his3*∆*200 leu2*∆*1 MCM21-13Myc:kanMX6* | This study |
| Y1709 | *MAT***a** *ura3-52 lys2-801 ade2-101 trp1*∆*63 his3*∆*200 leu2*∆*1 MTW1-13Myc:kanMX6* | This study |
| Y1710 | *MAT***a** *ura3-52 lys2-801 ade2-101 trp1*∆*63 his3*∆*200 leu2*∆*1 NSL1-13Myc:kanMX6* | This study |
| Y1711 | *MAT***a** *ura3-52 lys2-801 ade2-101 trp1*∆*63 his3*∆*200 leu2*∆*1 NNF1-13Myc:kanMX6* | This study |
| Y1712 | *MAT***a** *ura3-52 lys2-801 ade2-101 trp1*∆*63 his3*∆*200 leu2*∆*1 DSN1-13Myc:kanMX6* | This study |
| Y1713 | *MAT***a** *ura3-52 lys2-801 ade2-101 trp1*∆*63 his3*∆*200 leu2*∆*1 NDC80-13Myc:kanMX6* | This study |
| Y1714 | *MAT***a** *ura3-52 lys2-801 ade2-101 trp1*∆*63 his3*∆*200 leu2*∆*1 NUF2-13Myc:kanMX6* | This study |
| Y1715 | *MAT***a** *ura3-52 lys2-801 ade2-101 trp1*∆*63 his3*∆*200 leu2*∆*1 SPC24-13Myc:His3MX6* | This study |
| Y1716 | *MAT***a** *ura3-52 lys2-801 ade2-101 trp1*∆*63 his3*∆*200 leu2*∆*1 SPC25-13Myc:kanMX6* | This study |
| Y1717 | *MAT***a** *ura3-52 lys2-801 ade2-101 trp1*∆*63 his3*∆*200 leu2*∆*1 SPC105-13Myc:His3MX6* | This study |
| Y1720 | *MAT***a** *ura3-52 lys2-801 ade2-101 trp1*∆*63 his3*∆*200 leu2*∆*1 SLK19-13Myc:His3MX6* | This study |
| Y1723 | *MAT***a** *ura3-52 lys2-801 ade2-101 trp1*∆*63 his3*∆*200 leu2*∆*1 IPL1-13Myc:His3MX6* | This study |
| Y1837 | *MAT***a** *ura3-52 lys2-801 ade2-101 trp1*∆*63 his3*∆*200 leu2*∆*1 SPC25-13Myc:kanMX6 MTW1-3HA:TRP1* | This study |
| Y1838 | *MAT***a** *ura3-52 lys2-801 ade2-101 trp1*∆*63 his3*∆*200 leu2*∆*1 ndc10-1 YBP2-13Myc:kanMX6* | This study |
| Y1839 | *MAT***a** *ura3-52 lys2-801 ade2-101 trp1*∆*63 his3*∆*200 leu2*∆*1 ybp2*∆*::His3MX6 SPC25-13Myc:kanMX6 MTW1-3HA:TRP1* | This study |
| Y1840 | *MAT***a** *ura3-52 lys2-801 ade2-101 trp1*∆*63 his3*∆*200 leu2*∆*1 ybp2*∆*::His3MX6 MTW1-13Myc:kanMX6* | This study |
| Y1841 | *MAT***a** *ura3-52 lys2-801 ade2-101 trp1*∆*63 his3*∆*200 leu2*∆*1 ybp2*∆*::His3MX6 NDC80-13Myc:kanMX6* | This study |
| Y1842 | *MAT***a** *ura3-52 lys2-801 ade2-101 trp1*∆*63 his3*∆*200 leu2*∆*1 ybp2*∆*::His3MX6 CTF19-13Myc:kanMX6* | This study |
| Y1866 | *MAT***a** *ura3-52 lys2-801 ade2-101 trp1*∆*63 his3*∆*200 leu2*∆*1 ybp2*∆*::His3MX6 MCM21-13Myc:kanMX6* | This study |
| Y1867 | *MAT***a** *ura3-52 lys2-801 ade2-101 trp1*∆*63 his3*∆*200 leu2*∆*1 ybp2*∆*::His3MX6 NUF2-13Myc:kanMX6* | This study |
| Y1868 | *MAT***a** *ura3-52 lys2-801 ade2-101 trp1*∆*63 his3*∆*200 leu2*∆*1 ybp2*∆*::His3MX6 DSN1-13Myc:kanMX6* | This study |
| Y899 | *MAT***a** *ura3-52 lys2-801 ade2-101 trp1*∆*63 his3*∆*200 leu2*∆*1 ctf8*∆*::TRP1 PDS1-13Myc:His3MX6* | This study |
| Y1858 | *MAT***a** *ura3-52 lys2-801 ade2-101 trp1*∆*63 his3*∆*200 leu2*∆*1 ctf8*∆*::TRP1 ybp2*∆*::kanMX6 PDS1-13Myc:His3MX6* | This study |
| YPH501 | *MAT***a**/*MAT*α *ura3-52/ura3-52 lys2-801/lys2-801 ade2-101/ade2-101 trp1*∆*63/ trp1*∆*63 his3*∆*200/ his3*∆*200 leu2*∆*1/ leu2*∆*1* | [53] |
| Y1847 | *MAT***a**/*MAT*α *ura3-52/ura3-52 lys2-801/lys2-801 ade2-101/ade2-101 trp1*∆*63/ trp1*∆*63 his3*∆*200/ his3*∆*200 leu2*∆*1/ leu2*∆*1 ybp2*∆*::kanMX6/ ybp2*∆*::kanMX6* | This study |
| SBY818 | *MAT***a** *ura3-1 leu2,3-112 his3-11:pCUP1-GFP12-lacI12:HIS3 trp1-1:lacO:TRP1 ade2-1 can1-100 bar1∆ lys2∆ PDS1-myc18:LEU2* | [35] |
| Y1859 | *MAT***a** *ura3-1 leu2,3-112 his3-11:pCUP1-GFP12-lacI12:HIS3 trp1-1:lacO:TRP1 ade2-1 can1-100 bar1∆ lys2∆ ybp2*∆*::kanMX6 PDS1-myc18:LEU2* | This study |
| 17/14 | *MAT***a** *his1* | [16] |
| 17/17 | *MAT*α *his1* | [16] |
